# Economic Considerations for Advancement Through the Progressive Control Pathway: Cost–Benefit Analysis of an FMD Disease-Free Zone in Punjab Province, Pakistan

**DOI:** 10.3389/fvets.2021.703473

**Published:** 2021-08-18

**Authors:** Nicholas A. Lyons, Muhammed Afzal, Farrukh Toirov, Aamer Irshad, Chris J. M. Bartels, Jonathan Rushton

**Affiliations:** ^1^Institute of Infection, Veterinary and Ecological Sciences, University of Liverpool, Liverpool, United Kingdom; ^2^European Commission for the Control of Foot-and-Mouth Disease, Food and Agriculture Organization of the United Nations, Rome, Italy; ^3^Food and Agricultural Organization of the United Nations, Islamabad, Pakistan; ^4^Animal Health Works, Bakhuizen, Netherlands

**Keywords:** cost-benefit analysis, foot-and-mouth disease, disease free zone, economic impact, progressive control pathway, vaccines

## Abstract

Foot-and-mouth disease (FMD) is a priority disease of livestock in Pakistan, which was classified in stage 2 of the Progressive Control Pathway (PCP-FMD) in 2015, aiming to reduce disease impact. Further progression requires efforts to reduce viral circulation that may ultimately result in being awarded official disease-free status by the World Organisation for Animal Health [Office International des Epizooties (OIE)]. Typically, FMD control is reliant on the extensive use of vaccines, requiring careful consideration of the costs and benefits to ensure investment is likely to provide a positive return. This study conducted a cost–benefit analysis (CBA) for a proposed zone within Punjab Province, Pakistan. Benefits were assumed to come from averted production losses and treatment costs and the costs based on typical measures required for establishing a disease-free zone. To estimate the impact of FMD at the farm level, models were created to estimate effects on milk production, offtakes, and changes in herd value over a 5-year period with different parameters used to represent the production systems present. Control strategy costs incorporated aspects of vaccination, surveillance, sanitary measures, program management, stakeholder engagement, preparatory studies, training, and capacity building. The results indicated a median benefit–cost ratio of 1.03 (90% central range 0.37, 1.63) with a median net present value of 1.99 billion Pakistan Rupees (90% central range −37.7, 37.0). The greatest cost was due to vaccination at 56%, followed by sanitary measures (including implementing and maintaining an animal ID system and quarantine stations around the zone) at 41%. Although the median benefit–cost ratio and net present value indicated that investment is likely to generate a positive return, the large variation indicates caution in interpreting the results and it is possible that an increase in animal value through new export markets will be required. Further refinement in our knowledge of disease impact and the details of the control strategy are needed. Moreover, there are implications regarding vaccine security, since the strategy is reliant on the steady provision of quality vaccines in order to achieve the anticipated benefits, raising important issues on vaccine availability for countries to maintain lucrative export markets for FMD.

## Introduction

Foot-and-mouth disease (FMD) is a highly contagious viral infection of livestock endemic throughout most of sub-Saharan Africa and large parts of Asia. The annual global economic cost of FMD has been estimated between US$6.50–21.0 billion in endemic regions alone with disproportional impacts on poor farmers dependent on livestock for incomes and food security (1). FMD is a World Organisation for Animal Health [Office International des Epizooties (OIE)]-listed disease (2), and its presence negatively impacts trade in animals and their products at national and international levels. For these reasons, there is huge expenditure in the control of FMD particularly through the use of vaccines that is estimated at over 2.3 billion doses annually (3).

In 2013, the Progressive Control Pathway for Foot-and-Mouth Disease (PCP-FMD) was launched by the European Commission for the Control of Foot-and-Mouth Disease (EuFMD), Food and Agriculture Organization (FAO), and OIE as a stepwise tool to assist endemic countries progressively reduce the impact of disease and virus circulation with the possibility of achieving a disease-free status (with or without vaccination) endorsed by the OIE (4). In PCP-FMD stage 1, activities are undertaken to understand the epidemiology and impact of FMD alongside an analysis of production systems and value chains to identify risk hotspots and target control measures to reduce the disease impact. These activities culminate in the creation of a Risk-Based Control Plan (RBSP) to enter PCP-FMD stage 2 aimed at reducing the impact. Progression to PCP-FMD stage 3 involves the creation of an Official Control Programme (OCP) aiming to reduce virus circulation that involves a significant increase in investment and demonstrates an intention to progress further toward FMD freedom and associated trade benefits.

The PCP-FMD is not prescriptive; countries take their own decisions on FMD mitigation measures to apply at national or zonal levels. It is recommended that decisions on implementation and progression should be supported by economics including baseline studies on disease impact and demonstrable benefits through rigorous monitoring and evaluation of the strategy. Due to the high additional cost associated with entering PCP stage 3, it is critical that an economic assessment is undertaken to ensure resources are appropriately allocated with a long-term vision to achieve disease freedom. Cost–benefit analysis (CBA) is an economic approach that is appropriate for assessing such long-term investments through adding the “time value of money” with costs and benefits occurring at different time points (5).

Pakistan is a large country within southern Asia with a sizable livestock population. Agriculture makes up approximately 18.5% of the national gross domestic product (GDP), with livestock being the largest contributor at approximately 60.5% (6). The livestock sector has been previously identified as playing an extremely important role in the rural economy, with improved production having the potential to alleviate poverty and contributing to household food security (7).

Livestock diseases are a major source of production loss in Pakistan, with FMD having been identified as a major contributor through impacts on milk production, fertility, and animals being sold at a reduced value (8), in addition to holding back potentially lucrative export markets to other countries. Pakistan is endemic for FMD, with three serotypes (A, O, and Asia-1) being commonly identified (9). It is actively engaged in the PCP-FMD reaching stage 2 in 2015, aiming to reduce the impact of disease in the dairy sector (10).

In recent years, FMD control has been particularly focused on Punjab province, the most populous province in Pakistan for both people and livestock, with relatively developed agricultural systems (11). In February 2020, a workshop in Pakistan, facilitated by the authors of this study and supported by FAO, indicated considerable interest in the creation of an export zone in Bahawalpur Division. This division lends itself to enhanced FMD control through natural barriers to the east (Cholistan Desert and border with India) and west (Sutlej River). The objective of this study was to use the outcomes of this workshop to undertake a CBA for PCP-FMD progression and establish a disease-free zone with vaccination in Bahawalpur Division.

## Materials and Methods

### General Approach

The CBA assumed benefits in the form of averted production losses and treatment costs among different systems in the proposed control zone and buffer area. At the time of the analysis, the potential benefits from exports were not quantified, so they would be considered in the form of a break-even analysis to achieve a benefit–cost ratio (BCR) of >1.0 (or net present value >0) if the BCRs based on averted production losses and treatment costs were <1.0.

The costs of the strategy were based on the assumption that the first 2 years would involve development of the OCP entering PCP stage 3 at the end of this period. The OCP would be implemented over the following 3 years with eventual OIE endorsement, allowing the zone to enter PCP stage 4. The categories of costs considered in the strategy were as follows:

- Vaccination and post-vaccination monitoring (PVM)
- Surveillance
  ◦ Passive–outbreak investigation
  ◦ Active–serosurveillance and surveillance for clinical disease
- Sanitary measures
  ◦ Implementation of an animal identification system
  ◦ Inspection posts around zone
- Program management, stakeholder engagement, and communications
- Preparatory studies, training, and capacity building.

These costs were estimated including the proposed zone and a 10–20-km buffer area. The initial costs of the program components were estimated by the authors with assumptions checked with experts from Pakistan facilitated by FAO-Pakistan and updated accordingly.

The analysis was performed over a 20-year period, with year 0 being the current FMD situation in the proposed zone. All models were developed in RStudio (12) with variability and uncertainty incorporated using Monte Carlo simulations in the mc2d package (13). Parametric distributions were fit using the fitdistrplus and propagate packages (14, 15). For converting estimates to US$, an exchange rate of 165 PKR/USD was used throughout.

### Description of Proposed Zone

**Figure 1:**
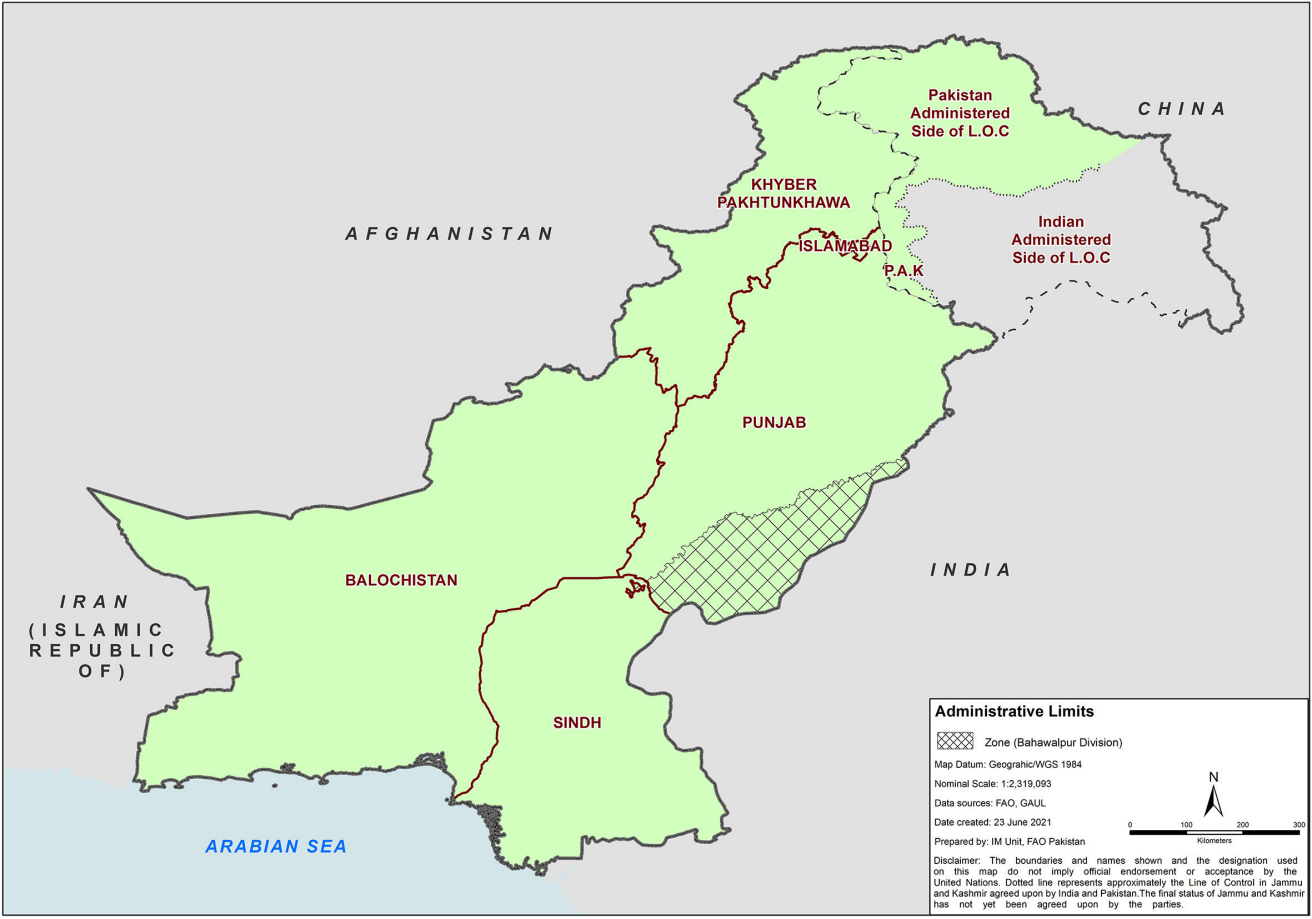
Geographical representation of the perceived foot-and-mouth disease (FMD) control zone in Punjab province bordered by the Sutlej River, Sindh province, and India. The zone encompasses the entirety of Bahawalpur Division, with the Sutlej River forming the Northern barrier of the zone between the zone and the rest of Punjab province.

The proposed zone for this analysis was located in Bahawalpur Division, southern Punjab. The geographical boundaries of this zone are represented in Figure 1. A 10–20-km buffer area was proposed to run alongside the edges of the zone within Pakistan. Within the buffer area, enhanced surveillance and control would take place to minimize the probability of incursions into the free zone. The eastern edge was bordering India that was considered impermeable to animal movements due to the presence of a high-security fence that is between ~1.8 and 2.4 m high, with circular blade wire on the top and 15-cm^2^ cement poles. The Sutlej River was considered to act as an effective geographical barrier between Bahawalpur Division and other divisions in Punjab province. There were an estimated 15 bridges over which animal movements may occur, and movements not *via* a bridge were considered extremely unlikely. The southern border of the zone alongside Sindh province is considered a high-risk area requiring additional efforts to restrict animal movements and ensure effective biosecurity. In both the proposed zone and buffer area, wild pigs are present, estimated at a few thousand and restricted to jungle areas. Occasionally, they have been seen among crops, but contact with local FMD-susceptible livestock is considered very rare but cannot be ruled out. Within Bahawalpur Division, Chinkara and Nilgai are also present, estimated at ~6,500 and <100, respectively, and restricted to the Lal Suhanra National Park that has a surrounding wall restricting contact with livestock.

### Benefits of Foot-and-Mouth Disease Control Zone and Buffer Area

#### Estimating the Impact of Foot-and-Mouth Disease in Production Systems

For estimating the impact of an FMD outbreak on farm production, stochastic farm models were created to account for the variability in farm types, management practices, and uncertainty in the model parameters. Separate general models were created for dairy and beef farming systems and parameterized based on data from the academic and gray literature, expert opinion, and assumptions (Supplementary Material A). Due to the variety of dairy systems in Pakistan, with different emphases on dairy production, this model was referred to as “mixed systems.”

##### Dairy “Mixed” Systems

**Figure 2:**
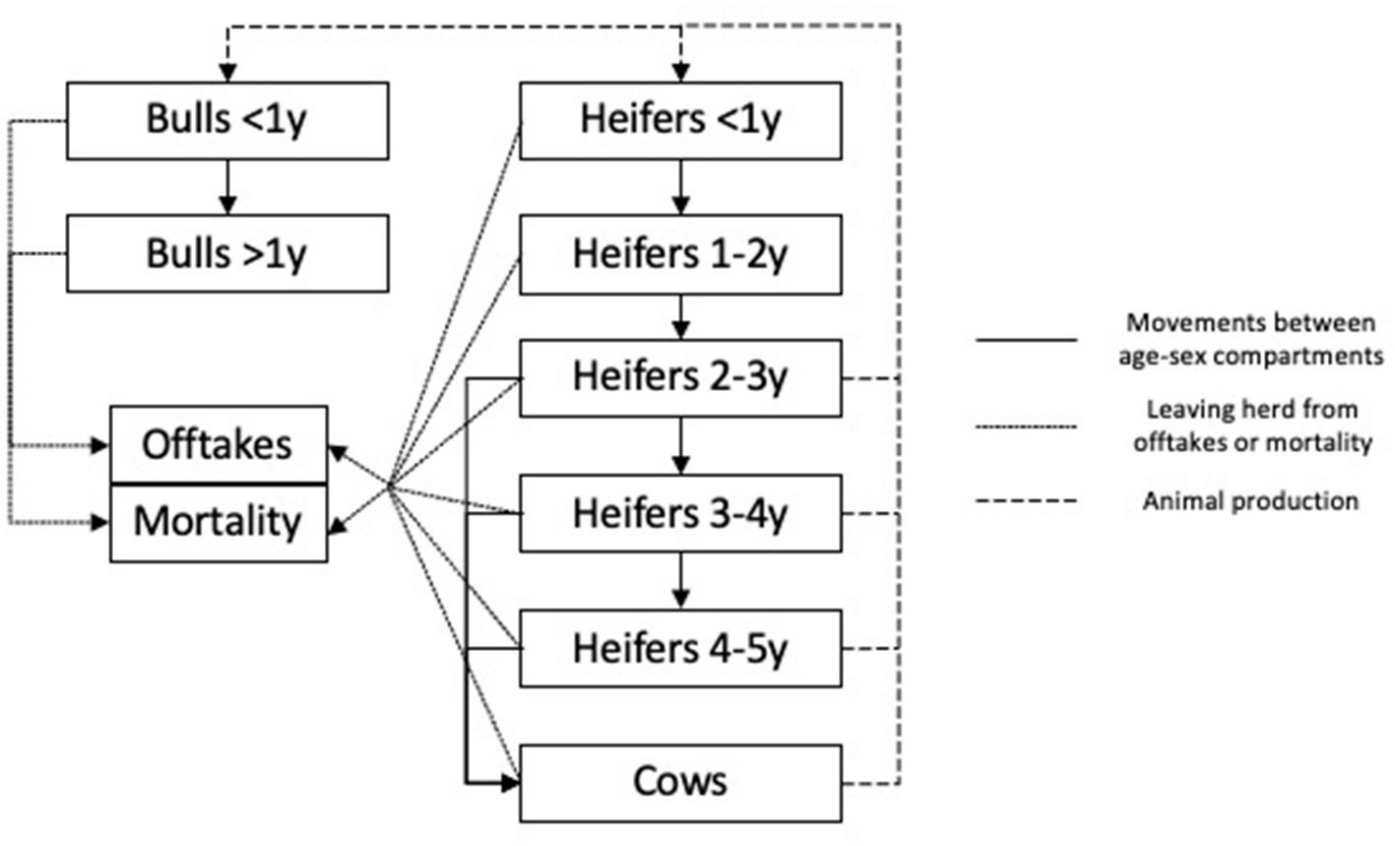
Model framework representing the relationships between annual age–sex compartments for mixed-farming systems considered in the study. Each model was run over a 5-year period.

For mixed systems, farm outputs were compared between a non-affected farm and one affected with FMD. A generic mixed system model was created that incorporated yearly age–sex compartments with simulations over a 5-year period to account for the longer terms impact of FMD and changes in herd structure (Figure 2). Within this generic model framework, parameters were used to reflect the different farming systems present in the proposed zone and buffer areas (16, 17). The systems considered were smallholder subsistence, smallholder market-oriented, rural commercial, peri-urban, corporate, and desert farming. All data used on the production systems are provided in the Supplementary Materials. The distinction between these systems is based on a combination of size and production objectives based on the definitions of de Jong (16). Smallholder subsistence farms were focused on meeting family requirements at minimal costs, whereas market-oriented farms were larger, aiming to contribute to the milk market. Rural commercial farms are larger still and have invested in milk production but access similar milk markets to smallholders. Peri-urban farms are similar in size or larger than the rural commercial farms, but milk is sold to retail or intermediaries. Corporate farms are significantly larger, only use cattle, and have invested even more in production with advanced animal genetics and facilities. Finally, the desert farms have a variable size and are transhumant, as described by (18). Based on the previous vaccination campaigns, there were 127,289 smallholder subsistence farms, 280,699 market-oriented, 725 rural commercial, 347 peri-urban, and 19,251 desert livestock farms. For the corporate farms, there were only two in the proposed zone (one with ~3,500 head and the other 200 head) and none in the buffer area, so these farms were parameterized separately with fixed herd sizes (Supplementary Table A1).

In year 1 of the simulation, the model derived a farm size from the relevant distribution for each production system and divided the farm population into animals with and without disease, and separate parameters were used to reflect the different fertility rates, mortality, and offtakes (i.e., voluntary and nonvoluntary culling) (Supplementary Tables A2–4).

For cows, the fertility rate (defined by the proportion of cows producing a calf each year) was determined by a combination of voluntary waiting periods, heat detection rate, and pregnancy rates. The impact of FMD on fertility in cows was modeled through missed heats and abortions. The fertility rate in heifers was determined by the heat detection, pregnancy, and abortion rates. To account for the impact of FMD on the age of first calving, reported as being extended in FMD cases (19), the yearly number of births was distributed among different heifer age categories (i.e., 2–3, 3–4, and 4–5 years) according to proportions reported in the Pakistan Livestock Census (20). The extended age of first calving in FMD cases was estimated through expert opinion to give the Pakistan context and used to calculate how these proportions would change (i.e., an extended age of first calving would lead to a relatively higher proportion in older age categories).

All equations for estimating the size and composition of the age–sex compartments are shown in Table 1 with an explanation of the symbols in Table 2. The equations for age–sex distribution in subsequent years (years 3–5) are the same for year 2 only that non-case parameters were used. It was assumed that all heifers that did not calve by 5 years of age were culled (and included in the annual offtakes), and calves had an equal probability of being male or female.

**Table 1:** Model equations used in dairy “mixed” systems for estimating the composition of age–sex compartments for each year of the simulation.

**Year**	**Age–sex compartment**	**Equation**	**Number**
1	All	${Y1}_{c}^{x}=hs\;\times \;hp^{x}\times inc^{x}$	1
		${Y1}_{nc}^{x}=hs\;\times \;hp^{x}\times \;\left(1-inc^{x}\right)$	2
		${Y1}_{t}^{x}={Y1}_{nc}^{x}+\;{Y1}_{c}^{x}$	3
2	Calves <1 year old	$Y2_{y}^{cfx}=\left(\begin{array}{l} \frac{(Y1_{y}^{c}\;\times \left(1-C_{y}^{c}\right)\;\times \;\left(1-M_{y}^{c}\right)\;\times \;F_{y}^{c})+\;}{2}\\ +\;\sum_{z=2}^{4}\frac{Y1_{y}^{z-1}\times \left(1-C_{y}^{z-1}\right)\;\times \;\left(1-M_{y}^{z-1}\right)\;\times F_{y}^{z-1}}{2} \end{array}\right)$	4
		${Y2}_{t}^{cfm}=\;{Y2}_{c}^{cfm}+\;{Y2}_{nc}^{cfm}$	5
	Bulls >1 year old and heifers aged	${Y2}_{t}^{cff}=\;{Y2}_{c}^{cff}+\;{Y2}_{nc}^{cff}$	6
	1–2 years	${Y2}_{y}^{m1,h1}=\;{Y1}_{y}^{cfx}\times \;\left(1-C_{y}^{cfx}\right)\;\times \;\left(1-M_{y}^{cfx}\right)\times \;\left(1-D_{c}^{cfx}\right)$	7
		${Y2}_{t}^{m1}=\;{Y2}_{c}^{m1}+\;{Y2}_{nc}^{m1}$	8
	Heifers aged 2–5 years	${Y2}_{t}^{h1}=\;{Y2}_{c}^{h1}+\;{Y2}_{nc}^{h1}$	9
		${Y2}_{y}^{z}=\;\left({Y1}_{y}^{z-1}\times \;\left(1-C_{y}^{z-1}\right)\times \left(1-M_{y}^{z-1}\right)\times \;\left(1-D_{c}^{z-1}\right)\right)\times \left(1-F_{y}^{z-1}\right)$	10
	Cows	${Y2}_{t}^{z}=\;{Y2}_{c}^{z}+\;{Y2}_{nc}^{z}$	11
		$\begin{array}{l} Y2_{y}^{c}=\;(Y1_{y}^{c}\times \left(1-C_{y}^{c}\right)\;\times \;\left(1-M_{y}^{c}\right)\;\times \;\left(1-D_{c}^{c}\right))\\ +\sum_{z=2}^{4}Y_{y}^{z-1}\times \left(1-C_{y}^{z-1}\right)\;\times \;\left(1-M_{y}^{z-1}\right)\;\times F_{y}^{z-1}\# \end{array}$	12
3–5	Calves <1 year old	${Y2}_{t}^{c}=\;{Y2}_{c}^{c}+\;{Y2}_{nc}^{c}$	13
	Bulls >1 year old and heifers aged 1–2 years	$Y3\ldots 5_{t}^{cfx}=\left(\begin{array}{l} \frac{(Y2\ldots 4_{t}^{c}\;\times \left(1-C_{nc}^{c}\right)\;\times \;\left(1-M_{nc}^{c}\right)\;\times \;F_{nc}^{c})+\;}{2}\\ +\;\sum_{z=2}^{4}\frac{Y2\ldots 4_{t}^{z-1}\times \left(1-C_{nc}^{z-1}\right)\;\times \;\left(1-M_{nc}^{z-1}\right)\;\times F_{nc}^{z-1}}{2} \end{array}\right)$	14
	Heifers aged 2–5 years	${Y3\ldots 5}_{t}^{m1,h1}=\;{Y2\ldots 4}_{t}^{cfx}\times \;\left(1-C_{nc}^{cfx}\right)\;\times \;\left(1-M_{nc}^{cfx}\right)$	15
		${Y3\ldots 5}_{t}^{z}=\;\left({Y2\ldots 4}_{t}^{z-1}\times \;\left(1-C_{nc}^{z-1}\right)\times \left(1-M_{nc}^{z-1}\right)\right)\times \left(1-F_{nc}^{z-1}\right)$	16
	Cows	${Y3\ldots 5}_{t}^{z}=\;\left({Y2\ldots 4}_{t}^{z-1}\times \;\left(1-C_{nc}^{z-1}\right)\times \left(1-M_{nc}^{z-1}\right)\right)$ + $\overset{4}{\underset{z=2}{\sum }}{Y2\ldots 4}_{t}^{z-1}\times \left(1-C_{nc}^{z-1}\right)\;\times \;\left(1-M_{nc}^{z-1}\right)\;\times F_{nc}^{z-1}$	17

*See Table 2 for an explanation of the equation symbols. Superscript reflects age–sex category, while subscript reflects whether they are cases, non-cases, or the total number in each compartment*.

**Table 2:** Explanation of model symbols for estimating the impact of foot-and-mouth disease (FMD) on a farm in terms of fertility, herd structure, and milk yield.

**Symbol**	**Explanation**	**Data source**
${Y1}_{c}^{x}$	Number of FMD cases in Year 1	Calculated (see Table 1)
${Y1}_{nc}^{x}$	Number of non-cases in Year 1	Calculated (see Table 1)
${Y1}_{t}^{x}$	Total herd size in Year 1	Calculated (see Table 1)
hs	Assumed herd size at start of the simulation	(16, 21–25)
hp^x^	Proportion in each age-production category (*x*)	
inc^x^	Incidence of FMD in each age-production category (*x*)	(21)
$F_{y}^{c}$	Fertility rate in cows	Calculated (see Table 3)
$C_{y}^{c}$	Culling/offtake rates in cows	Expert opinion
$M_{y}^{c}$	Mortality rate in cows	Expert opinion
$D_{c}^{c}$	Distress sales rate in cows	Expert opinion
$F_{y}^{z-1}$	Fertility rates among heifers in the previous year compartment	Calculated (see Table 3)
$C_{y}^{z-1}$	Culling/offtake rates among heifers in the previous year compartment	Expert opinion
$M_{y}^{z-1}$	Mortality rates among heifers in the previous year compartment	Expert opinion
$D_{c}^{z-1}$	Distress sales rate among heifers in the previous year compartment	Expert opinion
$C_{y}^{cfx}$	Culling/offtake rates in calves	Expert opinion
$M_{y}^{cfx}$	Mortality rate in calves	Expert opinion
$D_{y}^{cfx}$	Distress sales in calves	Expert opinion
$PR_{y}^{c}$	Pregnancy rate	Expert opinion
$HDR_{y}^{c}$	Heat detection rate	Expert opinion
*PL*	Proportion of cows lactating	Expert opinion
*dmy* ^*nc*^	Daily milk yield in non-cases	Expert opinion; (21)
*dmy* ^*cd*^ *or* *dmy* ^*cr*^	Daily milk yield in cases during the disease (cd) or recover (cr) phases	Expert opinion; (21)
*dmd* ^*d*^ *or dmd* ^*r*^	Duration of milk drop due to FMD (days) in the disease (d) or recovery (r) phases	(21), and author assumption
*vwp*	Voluntary waiting period	Expert opinion
*c_missed*	Number of cycles missed in a FMD case	Expert opinion
*abort_*c*_*	Abortion rate in FMD cases (cows)	Expert opinion
*abort_*z*_*	Abortion rate in FMD cases (heifers)	Expert opinion
*cfr*	Case fatality rate	Expert opinion
*tx*	Treatment cost (medicines per FMD case)	Expert opinion
*labour*	Labour cost (per minute)	Expert opinion
*tx_time*	Treatment time (minutes per FMD case)	Expert opinion
*fvalue*	Finished animal value	Expert opinion
*fvalue_*c*_*	Finished animal value (after FMD)	Expert opinion
*fvalue_*ds*_*	Proportion of animal value as distress sale	Expert opinion
*tof*	Time in feedlot (days)	Author assumption
*dfc*	Daily feed cost (PKR)	Expert opinion
*eft*	Extended finishing time (days for FMD case)	Expert opinion
*di*	Normal feed intakes (kg/day DM)	Expert opinion
*di_*c*_*	FMD case feed intakes (kg/day DM)	Expert opinion
*fc*	Feed cost (kg DM)	Expert opinion
*dfmd*	Duration of reduced intakes for FMD case (days)	
**Superscript and subscripts**		
x	Age-sex category	N/A
cfm	Male calves <1 year old	N/A
m1	Male calves >1 year old	N/A
cff	Female calves <1 year old	N/A
h1	Heifers aged 1–2 years old	N/A
h2	Heifers aged 2–3 years old	N/A
h3	Heifers aged 3–4 years old	N/A
h4	Heifers aged 4–5 years old	N/A
c	Cows – any animal that has had at least one calf	N/A
cfx	*cfm* or *cff* (see Table 2)	N/A
z	Heifer age category (h2, h3, h4)	N/A
y	Cases (c) or Non-cases (nc)	N/A
t	Total number of animals	N/A

The production outputs considered were yearly farm-level milk production, offtakes, and changes in herd value. Expert opinion was used to estimate the current market value of animals used for calculating the revenue from offtakes that were summed to estimate the herd value. In the first year of the simulation, during which the outbreak is assumed to have occurred, distress sales were also included with these animals having a lower value. Separate models were run for buffalo and cattle present in each farming system using species specific parameters, with the totals combined to give the overall impact in that farming system. Models also estimated the cost of treatment of clinical cases using reports from the literature.

Annual estimates of the impact of FMD on milk production was also calculated over the 5-year simulation, accounting for changes in herd structure. For year 1, this incorporated the reduced milk production in cases of FMD. Based on the report of Ferrari et al. (21), the estimated reduction in milk production for clinical FMD cases was based on “disease” and “recovery” phases using separate estimates of daily milk yields. The calculation for milk loss is represented in Table 3 (Equation 24) with symbols explained in Table 2.

**Table 3:** Equations for calculating fertility and milk yield parameters. See Table 2 for an explanation of the symbols.

**Parameter**	**Symbol**	**Equation**	**Number**
Fertility rate in cows (cases)	$F_{c}^{c}$	$1-\left(1-{\left(PR_{c}^{c}\times HDR_{c}^{c}\right)}^{cy_{c}}\right)-abort_{c}$	18
Fertility rate in cows (non-cases)	$F_{nc}^{c}$	$1-\left(1-{\left(PR_{nc}^{c}\times HDR_{nc}^{c}\right)}^{cy_{nc}}\right)$	19
Fertility rate in heifers (cases)	$F_{c}^{z}$	$1-\left(1-{\left(PR_{z}^{c}\times HDR_{z}^{c}\right)}^{nc_{cy}}\right)-abort_{z}$	20
Fertility rate in heifers (non-cases)	$F_{nc}^{z}$	$1-\left(1-{\left(PR_{z}^{c}\times HDR_{z}^{c}\right)}^{nc_{cy}}\right)$	21
Number of cycles in a year (non-case)	*cy_*nc*_*	$\frac{365-vwp}{21}$	22
Number of cycles in a year (case)	*cy_*c*_*	*cy_*nc*_ - c_missed*	23
Annual milk yield loss in liters (cases)	*ML*	$\begin{array}{l} Y1_{c}^{c}\;\times PL\;\times \;\left(dmy^{nc}\times \left(365-\left(dmd^{d}+dmd^{r}\right)\right)\right)\\ \;+\left(+\;\left(dmd^{d}\times dmy^{cd}\right)+\;\left(dmd^{r}\times dmy^{cr}\right)\right) \end{array}$	24

The cost of treatment for clinical cases of FMD was based on estimates from the literature and expert opinion (Supplementary Material A).

##### Beef Feedlot System

Based on data collected during the previous vaccination campaign, there are 155 beef feedlots in the proposed zone. The beef production system model was based on a 90-day fattening system considered typical for the current feedlot systems in the proposed zone. The model incorporated the following components in estimating the impact of an FMD outbreak: (1) cost of treatment of cases; (2) case fatality; (3) prolonged finishing time; (4) distress sales; and (5) reduced finishing value. Due to a lack of available data, estimates were made for case farms only rather than comparison with a non-affected equivalent as per the mixed-system model previously described.

In treating cases, the cost of labor was incorporated using daily rates and cost per minute calculated based on a 9-h working day (the maximum permitted under current Pakistani law). Case fatality was based on the final finished value of the animal minus the costs saved finishing the animal through provision of feed. It was assumed that there was an equal probability of having FMD throughout the time on the feedlot. Daily feed costs were estimated based on the range of intakes in feedlot systems and the cost per kilogram of feed.

Using information provided by feedlot farmers based in the proposed zone, two courses of action were reported to occur with animals that have FMD on feedlots. Either they are sold immediately as distress sales or they are kept on the feedlot for finishing but tend to require longer finishing times and have a lower final value. The model was created so that 40% of farms undertook distress sales, while 60% undertook the finishing option, consistent with the responses from the farmers who provided information on feedlots. For the finishing option, reduced feed intakes were assumed while sick, with the duration of illness assumed to last between 3 and 10 days. The extended time to finish the animals was based on expert opinion. For the distress sale option, it was assumed that the value of the animal was between 50 and 75% of the final finishing value. The costs saved from provision of feed were included as previously, with the assumption that there was an equal probability of having disease throughout the time on the feedlot.

The equations estimating the impacts among beef feedlots are shown in Table 4. All models incorporated variability and uncertainty through the use of stochastic parameters with all data and distributions provided in the Supplementary Material.

**Table 4:** Equations used to estimate the impact of foot-and-mouth disease (FMD) among beef feedlot systems.

**Cost component**	**Equation**	**Number**
Treatment of FMD cases	(*hs* × *inc*) × (*tx* + (*labour* × *tx*_*time*))	25
Case fatality	(*hs* × *inc* × *cfr* × *fvalue*) − ((90 − *tof*) × *dfc*)	26
Prolonged finishing time	(*hs* × *inc*) × ((*fvalue* − *fvalue*_*c*_) + ((*eft* × *dfc*) − (*dfmd* × (*di* − *di*_*c*_) × *fc*)))	27
Distress sales	(*hs* × *inc*) × (*fvalue* × *fvalue*_*ds*_) − ((90 − *tof*) × *dfc*)	28

*See Table 2 for an explanation of the symbols*.

#### Number of Outbreaks

To estimate the overall benefits of the zone, the number of outbreaks that would be expected to occur with no control measures in place was estimated, which forms the baseline scenario for comparison with the intervention. FMD is a notifiable disease in Pakistan. Since the vaccination campaign was initiated in 2018, the number of outbreaks for this baseline scenario was based on the typical range in the preceding years and included in the model as a stochastic parameter incorporating underreporting. A linear decline in the number of outbreaks was assumed with the intervention (Supplementary Table B1). Separate outbreak data were used for zone and buffer area (Supplementary Tables B1, 2, respectively). Detailed justification for the assumed number of outbreaks in the different systems is provided in Supplementary Material B.

### Costs of the Foot-and-Mouth Disease Control Strategy

The various costs of the control strategy are represented in the following sections with all data used provided in Supplementary Material C. For converting estimates to US$, an exchange rate of 165 PKR/USD was used.

#### Vaccination and Post-vaccination Monitoring

The cost of vaccination was based on a two-dose primary course for youngstock and 6-monthly boosters applied to all cattle and buffalo present in both the zone and buffer areas. The vaccine was expected to be imported, with an estimated 80% being aqueous adjuvanted and the remaining 20% being oil adjuvanted. Delivery costs were also included, and the required number of doses was based on previous campaigns in the region, with the population size estimated to remain constant for the analysis period.

PVM was assumed to include an annual small-scale immunogenicity study and an annual population immunity study using blood samples collected for active surveillance strategy (see Passive and Active Surveillance section). The protocols were assumed to be based on the recommendations given in the FAO-OIE PVM Guidelines (26) and local laboratory costs based on expert opinion.

Since the described vaccination policy was occurring at the time of the analysis, it was assumed to be a cost in year 0, although the costs of PVM was assumed to start in year 1. Both aspects were assumed to occur each year for the duration of the study (i.e., continues when officially free of FMD in the zone).

#### Passive and Active Surveillance

As recommended in the PCP-FMD guidelines for stage 3, it was assumed that all reported outbreaks of FMD were fully investigated. The cost model assumed two field staff would attend each outbreak, and samples would be taken and sent to the laboratory for virus testing as per usual laboratory procedures. It was also assumed that an annual shipment would be sent to an OIE reference laboratory where testing is conducted at zero cost to the country.

Each year, a serosurveillance study for non-structural protein (NSP) antibodies was assumed to take place with an appropriate sample size to demonstrate an NSP seroprevalence that declines each year implementing the disease control strategy. When free of FMD, anticipated in year 5 of the analysis, the sample size was based on demonstrating freedom from infection.

The active surveillance was also assumed to include live animal market inspections for clinical disease. Between 15 and 25 full-time veterinary officers were expected to be employed for this purpose whose roles would also be for outbreak investigations and serosurveillance activities in the control zone and buffer area.

#### Sanitary Measures

The sanitary measures included in the cost model included the establishment of an individual animal identification system and quarantine stations around the outside of the proposed zone at the border with the buffer area.

For the identification system, it was assumed that this would be based on plastic tags, tagging youngstock only with full implementation over a 5-year period. This system and the associated costs were based on those provided in Annex 2 of the FAO guidelines (27) with the year 5 costs assumed to be the yearly maintenance costs after full implementation of the system.

For the quarantine stations, it was assumed that these would be present at each of the 15 crossings over the Sutlej River, with 3–10 (most likely five) additional stations at the southern border of the zone with Sindh province. In year 1 of the cost model, it was assumed that there would be an initial outlay for consultancies and study tours to finalize the quarantine system, stations built in year 2 and staffed full time by two people, and annual maintenance costs from year 3 onward.

#### Program Management, Stakeholder Engagement, and Communications

To cover program management, it was assumed that there would be one full-time senior manager in charge, supported by three assistants beginning in year 1 of the analysis.

It was expected that a public–private partnership would need to be established to support the control strategy and to organize producers to take advantage of the potential beef export market. This was expected to require funds to initiate and support ongoing activities. Costs were also included to cover other meetings with stakeholders and for supporting communications. These costs were expected to be relatively higher during the first 2 years of the intervention.

#### Preparatory Studies, Training, and Capacity Building

Based on discussions with stakeholders in Pakistan, numerous preparatory studies were anticipated to finalize the details of the control strategy. This included consultants to undertake specific tasks, training to build capacity in the laboratory and elsewhere, and the development of preparedness and contingency plans. Moreover, since there was no export abattoir based in the proposed zone, it was proposed to construct this in year 2 of the strategy.

### Expert Opinion Elicitation

Through a workshop conducted in February 2020, expert opinion was provided to inform the model parameters that could not be parameterized from the academic and gray literature. A total of 38 attendees were present, being a combination of government and field veterinarians and farmers from the dairy and beef sector. The elicitation exercise was conducted by grouping the data needs into four categories as follows: Baseline production, FMD incidence, Impact of FMD cases, and Reaction and expenditure. The data requests provided to each group are provided in Supplementary Material D. Two groups were assigned per category, and discussions were held over the different parameters, with a consensus reached within the group. All data provided were utilized to estimate the parameters. A comparison was made between the groups, and a mean value was taken. Groups were asked to give a confidence rating (on a scale of 1–5) that was used for weighting the estimated values. If the estimated parameters differed by more than 10%, further clarity on the parameter was sought through correspondence with FAO-Pakistan.

During this initial workshop, numerous additional elements were discussed including the requirement for a better representation on beef feedlots and inclusion of desert livestock systems. To this end, data were sought after the workshop from five feedlot producers located within the proposed zone area. The data request is provided in Supplementary Material E. Farms were asked to provide minimum, most likely, and maximum values for the parameters. For the model inputs, these were combined, giving the inputs from the farms equal weighting. A similar exercise was undertaken for the desert livestock systems, which is provided in Supplementary Material F. This was based on the premise that these desert farms were parameterized the same as smallholder subsistence farms, and experts were asked to consider how they may differ by proposing alternative values for the model. This remote expert opinion elicitation was organized by FAO-Pakistan and was a substitute for the inability to conduct a follow-up workshop owing to the coronavirus (COVID-19) pandemic. For this reason, only four experts were available to provide input with the variability in responses incorporated into the parameters with all provided data being utilized.

### Cost–Benefit Analysis

The primary outputs of the CBA were the BCR and the net present value (NPV) as per the following equations:

(29) $$BCR=\frac{\sum \frac{Total\;benefits}{{\left(1+d\right)}^{y}}}{\sum \frac{Total\;costs}{{\left(1+d\right)}^{y}}}\;$$

(30) $$NPV=\sum \frac{Total\;benefits}{{\left(1+d\right)}^{y}}-\;\sum \frac{Total\;costs}{{\left(1+d\right)}^{y}}$$

where d = discount rate, and y = year of analysis.

The discount rate was included in the model as a uniform distribution allowed to vary between 3 and 4% due to the uncertainty in this parameter. The same discount rate was used for all benefits and costs. The analysis was conducted over a 20-year period, with 5,000 simulations to provide estimated minimum, median, and maximum values.

A sensitivity analysis of the final CBA was conducted to determine which stochastic elements had the largest impact on the model outcomes through generation of Spearman Rho statistics and tornado plots.

## Results

### Impact of Foot-and-Mouth Disease at the Farm Level

The impact of FMD outbreaks among the different farming systems was estimated with the results presented in Table 5. There was a large variation in the estimated losses among the different systems that can be largely attributed to the numbers of cattle and buffalo on the farms. The lowest impacts were among the smallholder farms at a median estimate of 197,000 PKR (US$1,196) and 449,000 PKR (US$2,722) in subsistence and market-oriented systems, respectively. By far, the largest losses were among the corporate farms. For the larger corporate farms (*n* = 3,500), the overall estimated cost of an outbreak of FMD over a 5-year period from production losses and decreased herd value was a median of 1,785 m PKR (range 1,116–2,847 m PKR) equivalent to US$10.8 m (range US$6.7–17.3 m). On the smaller corporate farms (*n* = 200), an outbreak was estimated to have a cost of 102 m PKR (range US$61.7–181m) equivalent to US$626 k (range US$374 k–US$1.1 m). In all the mixed systems, the greatest cost was the loss of herd value over the 5-year simulation period at between 75 and 87%, the lowest being for corporate farms and the highest for smallholder farming systems (see Supplementary Material G).

**Table 5:** Model outputs for production systems showing discounted costs for a foot-and-mouth disease (FMD) outbreak at the farm level (median and 25th/75th percentiles).

**Production system**	**Overall costs (thousand PKR)**			**Overall costs (USD)**		
	**Median**	**25%**	**75%**	**Median**	**25%**	**75%**
Smallholder subsistence	197	154	247	1,196	933	1,498
Smallholder - market oriented	449	373	536	2,722	2,264	3,249
Rural commercial	2,324	1,773	2,950	14,084	10,745	17,882
Peri-urban	10,802	6,982	15,844	65,467	42,318	96,022
Desert farms	2,259	1,475	3,227	13,693	8,942	19,560
Beef feedlot	1,759	1,313	2,323	10,660	7,960	14,080
Corporate farm 1 (*n* = 3,500)	178,850	162,200	196,400	10.8 m	9.8 m	11.9 m
Corporate farm 1 (*n* = 200)	102,000	92,400	112,700	626,000	560,000	682,800

*Except for beef feedlot systems, models were run over a 5-year period with outbreaks occurring during year 1 of the simulation with 5,000 iterations. Overall costs are discounted by 3–4% represented in the model as a uniform distribution. Beef feedlot systems were run for one production cycle*.

### Cost–Benefit Analysis

The non-discounted costs and benefits for each year of the program over a 20-year period are shown in Supplementary Material H. The breakdown of the overall discounted costs by the different categories is shown in Table 6. The greatest cost was due to vaccination and PVM at 56%, followed by sanitary measures (including implementing and maintaining an animal ID system as well as quarantine stations around the zone) at 40.5%.

**Table 6:** Overall discounted costs by different categories for establishing and maintaining a disease-free zone in Punjab province, Pakistan.

**Cost category**	**Median (min, max) cost million PKR**	**Percentage %**
Vaccination (including post-vaccination monitoring)	7,792 (6,794–9,779)	56.0
Surveillance	64.6 (52.4–82.8)	0.46
Sanitary measures	5,630 (4,919–6,586)	40.5
Program management	25.8 (21.5–31.6)	0.19
Preparatory studies, training, and capacity building	402 (371–439)	2.9

*Percentages are based on the median values*.

**Figure 3:**
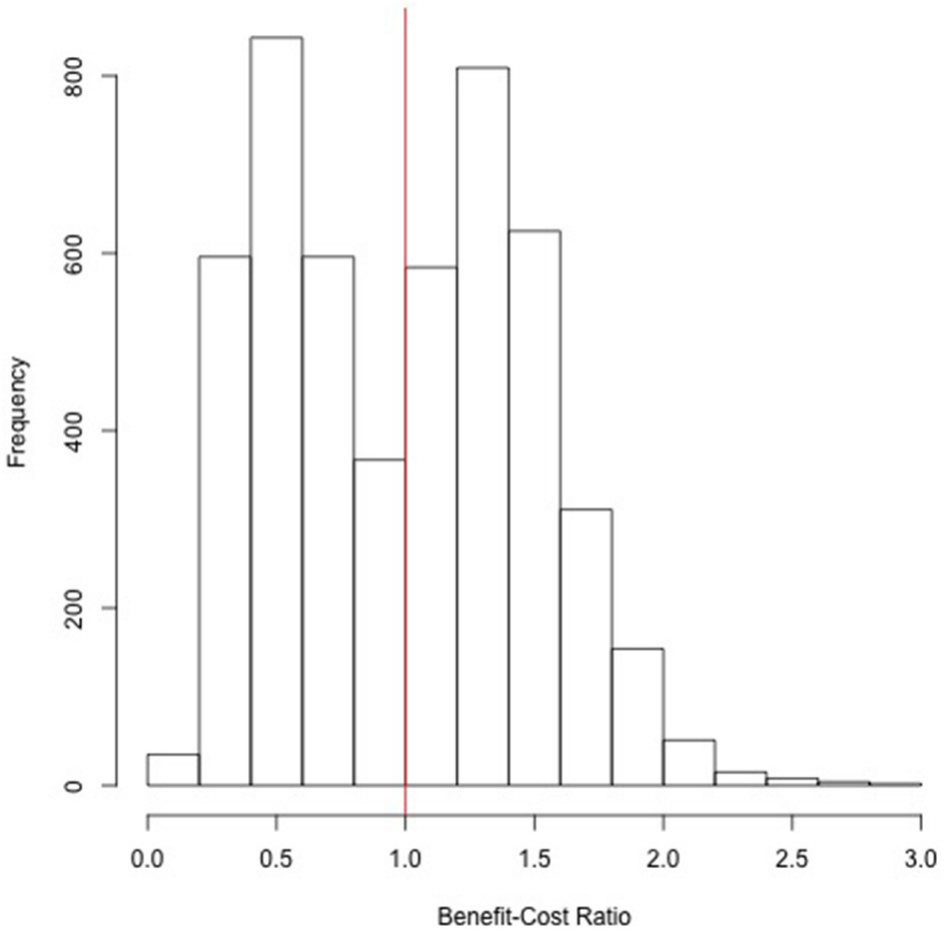
Histogram of benefit–cost ratio (BCR) estimates from the stochastic cost–benefit model based on 5,000 simulations. The vertical red line is at a BCR of 1 over which the investment is considered financially worthwhile.

The median BCR was estimated to be 1.03 (90% central range 0.37, 1.63) with a median NPV of 1.99 billion PKR (90% central range −37.7, 37.0). Without discounting, the median BCR was 1.01 (90% central range 0.37, 1.59). A histogram of the BCR values is shown in Figure 3 that shows a bimodal distribution that can be attributed to the high cost of outbreaks in the commercial farms (see Sensitivity Analysis).

### Sensitivity Analysis

**Figure 4:**
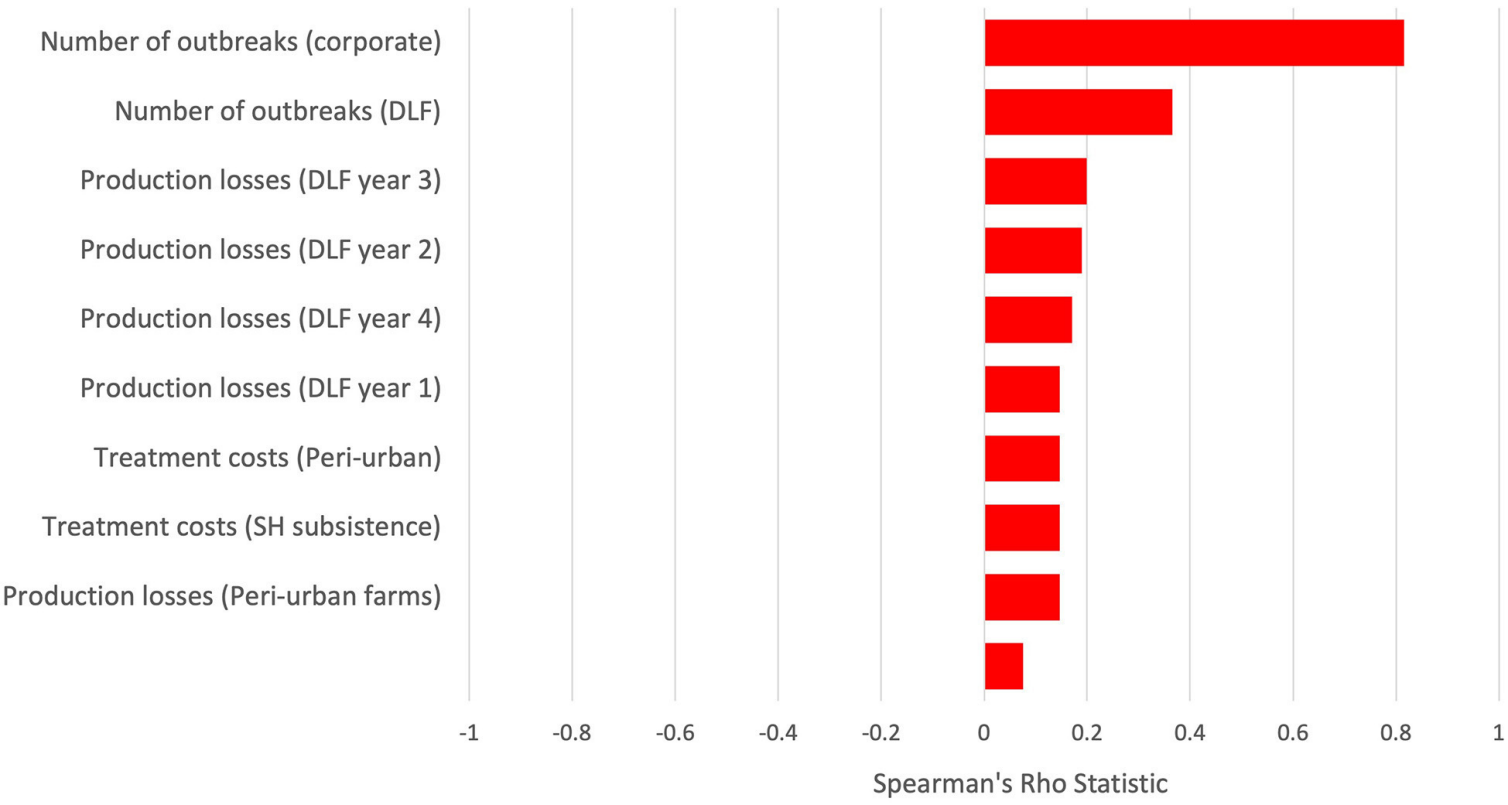
Sensitivity analysis–tornado plot demonstrating the top 10 variables affecting the results of the cost–benefit analysis.

Sensitivity analysis of the final CBA was conducted to demonstrate variables that had the largest impact on the overall BCR and inform areas where data collection may be more required to increase the precision in the estimates. Figure 4 is a tornado plot representing the top 10 parameters affecting the overall BCR based on the Spearman's Rho statistic. Positive values reflect parameters that increase the BCR and have a positive impact on the BCR.

As expected, the greater the number of outbreaks in the baseline scenario and the higher estimated farm level impacts, the greater the BCR, since there are greater losses averted with the control strategy. The sensitivity analysis indicates that, in the current model, the number of outbreaks in the corporate farms has the highest impact on the result owing to the huge losses seen in this farming system. The number of outbreaks in the desert livestock system also ranks highly that is likely due to the large degree of uncertainty in the number of outbreaks and impact in this system.

## Discussion

The analysis presented provides estimates on the relative costs and benefits of a proposed FMD-free zone in Bahawalpur Division, Punjab Province, Pakistan. The CBA indicated that with the historical levels of FMD and its impact on the existing production systems, increases in the activities to improve disease control and advance the zone toward elimination of the virus appear to be economically profitable, but there is a large degree of uncertainty and there are numerous aspects that need to be considered by policymakers when interpreting these results.

The highest farm-level losses were seen among the corporate farms that would potentially have the most to gain from control. The estimated farm-level losses due to FMD in the current study are substantially higher than previously published estimates from Pakistan. A relatively recent study estimated the overall financial impacts among rural livestock (equivalent to smallholders) and peri-urban farming systems at between 47,491 and 105,665 PKR for the former and 273,101 and 238,146 PKR for the latter (28). This is equivalent to ~25 and 5%, respectively, of the losses reported in the current study. These large differences may be explained in part by the longer-term losses considered in the current study. The previous study estimated that 81% of farm-level losses were due to a decrease in milk production compared to the current study that estimated these losses at between 5 and 20% depending on the farming system (Supplementary Material G). Although in this previous study approximately 40% of farmers reported a “disturbance” in calving intervals, the long-term economic consequence was not considered. Previous studies from Kenya have demonstrated that FMD was associated with an increase in the age of first calving, reduced conception rates (19), and increased culling several months after an outbreak occurs (29). Another study from Iran demonstrated reduced growth rates in dairy heifers (30) that can be associated with reduced lifetime productivity (31). Longer-term economic impacts of FMD at the farm level have received relatively little attention compared to short-term losses. One published example is among extensive beef systems in Bolivia that demonstrated that it can take 4–6 years for the economic impacts to manifest (5). With the typically high animal-level incidence of FMD, it is not surprising that impacts on fertility can lead to large reductions in herd-level productivity, although longer-term economic studies on affected farms would provide supportive evidence of these prolonged impacts.

There are numerous limitations to the study that should be highlighted and appreciated when considering the results. Firstly, the parameters used in the production system models were heavily reliant on expert opinion. Although the uncertainty in these estimates was accounted for in the analysis, this is in part responsible for the wide estimates in the BCR. The analysis would benefit from field studies being conducted in the proposed zone and buffer area. In particular, very little information was available on the epidemiology and economic impact of FMD among desert livestock farms that predominate in the Cholistan Desert region. One study indicated a 62.2% seroprevalence among cattle in Cholistan, although the analysis did not consider the incidence of disease at the farm level (32).

Secondly, an assumption that has a large impact was the number of outbreaks under baseline conditions (i.e., with no disease control in place) that were based on historic reporting. Again, for desert livestock farms, very few outbreaks were officially reported likely due to underreporting, and the large uncertainty in this parameter was reflected in the sensitivity analysis. Only limited expert opinion was made available for this parameter and no follow-up elicitation exercises were possible. Collection of data from this system using rigorous epidemiological design and analysis is therefore considered particularly important for refining the results of the analysis. An assumption of no further outbreaks occurring in the zone once declared free has not been considered in the approach, and if bordering areas remain endemic, this may be unlikely. The impacts of this scenario will be dependent on which export markets are being exploited and what plans are in place for dealing with them (for example, if stamping out would be utilized). The recent study published by (33) provides a useful approach for considering different policy options considering prospective market access and associated costs.

Finally, there are still details in the control strategy that need to be finalized, so that the costs can be more reliably estimated. Further studies are needed in particular to define the role of small ruminants in the epidemiology of FMD and whether they should be included in the vaccination strategy. Although the impact among small ruminant systems is often low due to mild or subclinical disease, this does not rule out a significant role in the transmission of virus at the population level. In Europe, FMD was eliminated through vaccinating large ruminants only, but the role of small ruminants in FMD epidemiology is likely to vary in different settings (34), and there are numerous studies in different parts of the world demonstrating significant seroprevalence (35–37). With an estimated small ruminant population close to 5 million in Bahawalpur Division, its inclusion in the vaccination campaign could be a considerable cost, so establishing its role in the epidemiology of FMD should be considered a priority particularly given the desire to be free of FMD within a 5-year period.

The greatest cost in the proposed control strategy was due to vaccines and vaccination. Vaccines are commonly used in the control of FMD and are considered essential for any endemic country contemplating advancement through the PCP-FMD. In this study, it was assumed that vaccines would be imported due to local vaccines being of insufficient quality (38) and that sufficient vaccine would be available to implement the strategy. Quality vaccines are difficult and costly to produce, and there is a global shortage in their availability (39). There are ongoing efforts from international agencies to promote the FMD Global Control strategy on which the PCP-FMD is the primary tool to support endemic countries (40). For this strategy to be successful, it is critical that there is sufficient supply of vaccines to meet the anticipated demand.

CBAs are commonly performed for evaluating disease control policy for FMD as summarized by Knight-Jones and Rushton (1). The likely returns on control are highly variable and depend heavily on the context and the export potential. Many of these studies were aimed at informing strategies if incursions were to occur in FMD-free countries. The establishment of FMD-free zones within endemic countries has received relatively little attention, and this is the first published study known to the authors that has considered an FMD-free zone in Pakistan. A similar study in Tanzania undertook a CBA for a zone in the Rukwa region that indicated that it was unlikely to be beneficial without clear benefits from exports (41). The results of the current analysis indicate an uncertain return on investment in establishing the free zone. Considering the lower range of estimated benefits, a substantial increase in the value of exports may be needed for the investment to break even and greater confidence in a return would come from export markets being clearly identified. There is a rapidly increasing global demand for meat, particularly in the halal market. However, according to a beef value chain analysis published in 2017, the current level of meat production in Pakistan is not sufficient to meet local demand despite rapid growth in meat production over the last several years (42). Moreover, meat consumption in Pakistan is relatively low by global standards and might be expected to increase. Higher beef exports to justify the advanced level of FMD control may lead to an even lower supply to the domestic market that may increase prices that may be unacceptable at a consumer and political level. Within Pakistan, it is estimated that 95% of meat is sold locally *via* “roadside” butchers (42). The single proposed control zone may not have an impact on national-level prices but could have an impact at a local level. Another wider aspect to consider is the benefits that may come from an increased capacity of the veterinary services to control other diseases [e.g., peste des petits ruminants (PPR)] and for dealing with emerging threats with implications for public health and food security. There are other social benefits and losses that may come from disease control, and these complex relationships could be explored using other economic approaches such as system dynamic models, so that the impact on the wider economy could be fully considered and captured (43–45). The inclusion of different stakeholders in the development of a control strategy will be important for effective implementation.

The discount rate used in the analysis reflects the opportunity cost of capital for the country in an agricultural context that the authors believe to be between 3 and 4%. If Pakistan seeks support from the development banks for the implementation of a new disease control strategy, it is likely that a rate of return on investment will be higher, possibly 8%. It is likely that the higher cost of capital would need to consider more carefully the stimulation of general investment in the livestock sector and the opportunity to access new markets. These elements of the wider economy would need further analysis to be fully captured.

## Data Availability Statement

The original contributions presented in the study are included in the article/Supplementary Material, further inquiries can be directed to the corresponding author/s.

## Author Contributions

NL led the study design, analysis, and writing of the manuscript with contributions from CB and JR. MA, AI, and FT led the conception of the study and facilitated the provision of expert opinion and other required data. All authors contributed to manuscript revision, read, and approved the submitted version.

## Conflict of Interest

The authors declare that the research was conducted in the absence of any commercial or financial relationships that could be construed as a potential conflict of interest.

## Publisher's Note

All claims expressed in this article are solely those of the authors and do not necessarily represent those of their affiliated organizations, or those of the publisher, the editors and the reviewers. Any product that may be evaluated in this article, or claim that may be made by its manufacturer, is not guaranteed or endorsed by the publisher.

## Supplementary Material

Supplementary Material A Production system data.

Supplementary Material B Number of outbreaks.

Supplementary Material C Program cost data.

Supplementary Material D Expert opinion exercise for workshop.

Supplementary Material E Data request for beef feedlots.

Supplementary Material F Expert opinion exercise for desert livestock systems.

Supplementary Material G Breakdown of impact by production system.

Supplementary Material H Results - costs and benefits by year.
