# Supplementary material for: Economic Considerations for Advancement Through the Progressive Control Pathway: Cost–Benefit Analysis of an FMD Disease-Free Zone in Punjab Province, Pakistan
Source: Front Vet Sci. 2021 Aug 18;8:703473. doi: 10.3389/fvets.2021.703473 (PMC8416473; doi:10.3389/fvets.2021.703473)
Supplement: Supplementary Material A — Production system data. [file Data_Sheet_1.zip › Supplementary material A - Production system data_updated.pdf]

Supplementary material A – Data used for production system models
Mixed-dairy production systems.

Table A1. **Dairy farming systems considered in the farm model including the starting model parameters. y=year. B = buffalo. C = cattle. The herd sizes were based on estimates provided in (de Jong, 2013; Farooq et al., 2007; Hagmann, 2011; Younas, 2013).**

Parameter	Category	Farming system										
		Smallholder – subsistence		Smallholder market - oriented		Rural Commercial		Peri-urban		Corporate	Desert	
		B	C	B	C	B	C	B	C	C	B	C
Herd size	Min	0.5	0.5	1.2	1.8	5	0	9	1	200	0	10
	Mode	1.5	1.5	2	3	27	3	45	5	1500	1.1	51
	Max	2.5	2.5	2.8	4.2	45	5	180	20	5000	5	200
Age-sex proportion	Bulls <1year	0.06	0.1	0.06	0.1	0.06	0.06	0.025	0.05	0.12	0.078	0.1
	Bulls >1y	0.02	0	0.02	0	0	0	0	0	0	0.017	0.002
	Heifers <1y	0.1	0.1	0.1	0.1	0.12	0.14	0.025	0.05	0.12	0.078	0.1
	Heifers 1-2y	0.09	0.1	0.09	0.1	0.09	0.1	0	0	0.1	0.042	0.022
	Heifers 2-3y	0.07	0.05	0.07	0.05	0.07	0.08	0.025	0.09	0.1	0.042	0.022
	Heifers 3-4y	0.05	0.05	0.05	0.05	0.05	0.05	0.025	0.09	0.06	0.042	0.022
	Heifers 4-5y	0.02	0	0.02	0	0.02	0.02	0.025	0.09	0	0.042	0.022
	Cows	0.59	0.6	0.59	0.6	0.59	0.55	0.875	0.63	0.5	0.66	0.71

Table A2. **Estimates of FMD morbidity on affected farms used in the production loss model based on that reported by Ferrari et al., 2013. n = total number of animals. Due to the absence of age-incidence data at the production system level, the same incidence data were used for each. B = buffalo. C = cattle.**

Age category	Category	Farming system										
		Smallholder subsistence		Smallholder market-oriented		Rural Commercial		Peri-urban		Corporate	Desert	
		B	C	B	C	B	C	B	C	C	B	C
<1 year	Incidence	0.56	0.7	0.56	0.7	0.56	0.74	0.56	0.74	0.74	0.56	0.74
	n	129	110	129	110	129	110	129	110	110	129	110
Heifers and males >1y	Incidence	0.5	0.7	0.5	0.7	0.5	0.7	0.5	0.7	0.7	0.5	0.7
	n	102	110	102	110	102	110	102	110	110	102	110
Cows	Incidence	0.43	0.6	0.43	0.6	0.43	0.56	0.43	0.56	0.56	0.43	0.56
	n	304	302	304	302	304	302	304	302	302	304	302

Table A3. **Mortality, offtake rates in FMD cases and non-cases, and distress sale rates in FMD cases by age-sex category for different dairy production systems. All figures are proportions. Distress sales reflect animals sold at a lower price so that a rapid sale can be made. B = buffalo. C = cattle.**

Parameter	Category	Farming system: Smallholder subsistence		Smallholder market-oriented		Rural Commercial		Peri-urban		Corporate	Desert		Data source
		B	C	B	C	B	C	B	C	C	B	C	
Male calves <1y													
Mortality rate (FMD cases)	Min	0.18	0.2	0.18	0.2	0.2	0.2	0.2	0.2	0.09	0.18	0.2	Expert opinion
	Mode	0.23	0.2	0.23	0.2	0.28	0.28	0.28	0.28	0.14	0.23	0.2	
	Max	0.4	0.4	0.4	0.4	0.45	0.45	0.45	0.45	0.23	0.4	0.4	
Mortality rate (non-cases)	Min	0.2	0.1	0.2	0.1	0.24	0.1	0.24	0.1	0.08	0.2	0.1	Expert opinion
	Mode	0.28	0.2	0.28	0.2	0.31	0.21	0.31	0.21	0.11	0.28	0.2	
	Max	0.4	0.3	0.4	0.3	0.39	0.31	0.39	0.31	0.16	0.4	0.3	
Offtake rates (FMD cases)	Min	0	0	0.05	0.1	0	0.05	0.05	0.05	0.4	0	0	Expert opinion
	Max	0.1	0.1	0.2	0.2	0.1	0.3	0.35	0.35	0.5	0.05	0.1	
Offtake rates (non-cases)	Min	0.3	0.3	0.3	0.3	0.5	0.5	0.5	0.5	0.75	0.3	0.9	Expert opinion
	Max	0.6	0.6	0.6	0.6	0.75	0.75	0.75	0.75	0.95	0.6	0.9	
Distress sales	Min	0	0	0.05	0.1	0.07	0.06	0.09	0.15	0.09	0	0	Expert opinion
	Max	0.08	0.1	0.23	0.2	0.28	0.34	0.47	0.55	0.36	0.08	0.1	
Female calves <1y													
Mortality rate (FMD cases)	Min	0.18	0.2	0.18	0.2	0.2	0.2	0.2	0.2	0.08	0.18	0.2	Expert opinion
	Mode	0.23	0.3	0.23	0.3	0.25	0.25	0.25	0.25	0.11	0.23	0.3	
	Max	0.4	0.4	0.4	0.4	0.4	0.4	0.4	0.4	0.2	0.4	0.4	
Mortality rate (non-cases)	Min	0.2	0.1	0.2	0.1	0.24	0.1	0.24	0.1	0.08	0.2	0.1	Expert opinion
	Mode	0.28	0.2	0.28	0.2	0.31	0.21	0.31	0.21	0.11	0.28	0.2	
	Max	0.4	0.3	0.4	0.3	0.39	0.31	0.39	0.31	0.16	0.4	0.3	
Offtake rates (FMD cases)	Min	0	0	0.05	0.1	0	0.05	0.05	0.05	0.4	0	0	Expert opinion
	Max	0.1	0.1	0.2	0.2	0.1	0.3	0.35	0.35	0.5	0.05	0.1	
Offtake rates (non-cases)	Min	0.05	0.1	0.05	0.1	0.08	0.08	0.08	0.08	0.28	0.05	0.15	Expert opinion
	Max	0.09	0.1	0.09	0.1	0.23	0.23	0.23	0.23	0.43	0.09	0.15	
Distress sales	Min	0	0	0	0	0	0.05	0.13	0.04	0.05	0	0	Expert opinion
	Max	0	0	0.04	0	0.04	0.15	0.47	0.22	0.52	0	0	
Bulls >1y													
Mortality rate (FMD cases)	Min	0	0	0	0	0.01	0.01	0.01	0.01	0.01	0	0	Expert opinion
	Mode	0.02	0	0.02	0	0.02	0.02	0.02	0.02	0.05	0.02	0	
	Max	0.04	0	0.04	0	0.04	0.04	0.04	0.04	0.09	0.04	0	
Mortality rate (non-cases)	Min	0.05	0.1	0.05	0.1	0.05	0.05	0.05	0.05	0.01	0.05	0.1	Expert opinion
	Mode	0.1	0.1	0.1	0.1	0.09	0.09	0.09	0.09	0.04	0.1	0.1	
	Max	0.15	0.1	0.15	0.1	0.13	0.13	0.13	0.13	0.07	0.15	0.1	
Offtake rates (FMD cases)	Min	0	0	0.02	0	0	0	0.05	0.03	0.4	0	0	Expert opinion
	Max	0.03	0	0.04	0	0.03	0.05	0.2	0.1	0.5	0.03	0	
Offtake rates (non-cases)	Min	0.65	0.7	0.65	0.7	0.75	0.75	0.75	0.75	0.45	0.65	0.9	Expert opinion
	Max	0.9	0.9	0.9	0.9	0.9	0.9	0.9	0.9	0.6	0.9	0.9	
Distress sales	Min	0.03	0	0.01	0	0.03	0.07	0.04	0.05	0.05	0.03	0	Expert opinion
	Max	0.29	0.3	0.12	0.1	0.14	0.19	0.31	0.25	0.5	0.29	0.3	
Heifers >1y													
Mortality rate (FMD cases)	Min	0	0	0	0	0.01	0.01	0.01	0.01	0	0	0	Expert opinion
	Mode	0.02	0	0.02	0	0.02	0.02	0.02	0.02	0.01	0.02	0	
	Max	0.04	0	0.04	0	0.04	0.04	0.04	0.04	0.03	0.04	0	
Mortality rate (non-cases)	Min	0.08	0.1	0.08	0.1	0.07	0.05	0.07	0.05	0.02	0.08	0.1	Expert opinion
	Mode	0.13	0.1	0.13	0.1	0.11	0.07	0.11	0.07	0.05	0.13	0.1	

Parameter	Category	Farming system											Data source
		Smallholder subsistence		Smallholder market-oriented		Rural Commercial		Peri-urban		Corporate	Desert		
		B	C	B	C	B	C	B	C	C	B	C	
	Max	0.18	0.1	0.18	0.1	0.15	0.1	0.15	0.1	0.07	0.18	0.1	
Offtake rates (FMD cases)	Min	0	0	0.03	0	0	0	0.05	0.05	0.4	0	0	Expert opinion
	Max	0.03	0	0.05	0.1	0.03	0.05	0.2	0.15	0.5	0.03	0	
Offtake rates (non-cases)	Min	0.08	0.1	0.08	0.1	0.08	0.08	0.08	0.08	0.08	0.08	0.1	Expert opinion
	Max	0.18	0.2	0.18	0.2	0.19	0.19	0.19	0.19	0.15	0.18	0.1	
Distress sales	Min	0	0	0	0	0	0.05	0.04	0.05	0.04	0	0	Expert opinion
	Max	0.07	0.1	0.04	0	0.04	0.15	0.24	0.2	0.47	0.07	0.1	
Cows													
Mortality rate (FMD cases)	Min	0	0	0	0	0	0	0	0	0	0	0	Expert opinion
	Mode	0	0	0	0	0.01	0.01	0.01	0.01	0	0	0	
	Max	0.01	0	0.01	0	0.03	0.03	0.03	0.03	0.01	0.01	0	
Mortality rate (non-cases)	Min	0.02	0	0.02	0	0.02	0.03	0.02	0.03	0.02	0.02	0	Expert opinion
	Mode	0.05	0.1	0.05	0.1	0.06	0.05	0.06	0.05	0.03	0.05	0.1	
	Max	0.09	0.1	0.09	0.1	0.09	0.08	0.09	0.08	0.04	0.09	0.1	
Offtake rates (FMD cases)	Min	0.04	0	0.05	0.1	0.07	0.1	0.18	0.18	0.45	0.04	0	Expert opinion
	Max	0.09	0.1	0.22	0.2	0.14	0.23	0.43	0.33	0.55	0.09	0.1	
Offtake rates (non-cases)	Min	0.03	0	0.03	0	0.04	0.04	0.04	0.04	0.03	0.03	0.05	Expert opinion
	Max	0.07	0.1	0.07	0.1	0.13	0.13	0.13	0.13	0.13	0.07	0.05	
Distress sales	Min	0.04	0	0.01	0	0.01	0.07	0.1	0.1	0.05	0.04	0	Expert opinion
	Max	0.09	0.1	0.12	0.1	0.08	0.19	0.29	0.22	0.53	0.09	0.1	

Table A4. **Fertility parameters used in model. B = buffalo. C = cattle.**

Parameter	Category	Farming system											Data source
		Smallholder subsistence		Smallholder market-oriented		Rural Commercial		Peri-urban		Corporate	Desert		
		B	C	B	C	B	C	B	C	C	B	C	
Pregnancy rate (cows)	Min	0.35	0.4	0.35	0.4	0.31	0.4	0.31	0.4	0.54	0.35	0.4	Expert opinion
	Max	0.65	0.6	0.65	0.6	0.56	0.6	0.56	0.6	0.78	0.65	0.6	
Heat detection rate (cows)	Min	0.4	0.3	0.4	0.3	0.48	0.34	0.48	0.34	0.5	0.4	0.3	Expert opinion
	Max	0.58	0.7	0.58	0.7	0.65	0.66	0.65	0.66	0.8	0.58	0.7	
Voluntary waiting period (days)	Min	90	60	90	60	95	60	95	60	55	90	60	Expert opinion
	Max	147	110	147	110	145	110	145	110	85	147	110	
Pregnancy rate (heifers)	Min	0.3	0.1	0.3	0.1	0.3	0.36	0.3	0.36	0.5	0.3	0.1	Expert opinion
	Max	0.6	0.7	0.6	0.7	0.6	0.71	0.6	0.71	0.74	0.6	0.7	
Heat detection rate (heifers)	Min	0.46	0.4	0.46	0.4	0.46	0.36	0.46	0.36	0.45	0.46	0.4	Expert opinion
	Max	0.66	0.7	0.66	0.7	0.66	0.7	0.66	0.7	0.8	0.66	0.7	
Missed cycles in FMD cases	Min	5	7.5	5	7.5	5	7.5	5	7.5	7.5	5	7.5	Expert opinion
	Mode	6.5	10.5	6.5	10.5	6.5	10.5	6.5	10.5	10.5	6.5	10.5	
	Max	11	16	11	16	11	16	11	16	16	11	16	

Parameter	Category	Farming system											Data source
		Smallholder subsistence		Smallholder market-oriented		Rural Commercial		Peri-urban		Corporate	Desert		
		B	C	B	C	B	C	B	C	C	B	C	
Abortion rate (cows)	Min	0.01	0.2	0.01	0.2	0.01	0.05	0.01	0.05	0.05	0.01	0.2	Export opinion
	Mode	0.03	0.1	0.03	0.1	0.03	0.08	0.03	0.08	0.08	0.03	0.1	
	Max	0.05	0.1	0.05	0.1	0.05	0.13	0.05	0.13	0.13	0.05	0.1	
Abortion rate (heifers)	Min	0.02	0.1	0.02	0.1	0.02	0.05	0.02	0.05	0.05	0.02	0.1	Expert opinion
	Mode	0.03	0.1	0.03	0.1	0.03	0.09	0.03	0.09	0.09	0.03	0.1	
	Max	0.08	0.1	0.08	0.1	0.08	0.15	0.08	0.15	0.15	0.08	0.1	
Cost of abortion (PKR)	Fixed	1500	1500	1500	1500	1500	1500	1500	1500	1500	1500	1500	(Fareed et al., 2017)
Age at first calving	2-3 years old	0.08	0.1	0.08	0.1	0.08	0.08	0.08	0.08	0.08	0.08	0.1	(Pakistan Bureau of Statistics, 2006)
	3-4 years old	0.78	0.8	0.78	0.8	0.78	0.78	0.78	0.78	0.78	0.78	0.8	
	4-5 years old	0.14	0.1	0.14	0.1	0.14	0.14	0.14	0.14	0.14	0.14	0.1	
Extended age of first calving (months)	Min	7	7.5	7	7.5	7	7.5	7	7.5	7.5	7	7.5	Expert opinion
	Mode	9	9	9	9	9	9	9	9	9	9	9	
	Max	12	12.5	12	12.5	12	12.5	12	12.5	12.5	12	12.5	

Table A5. **Estimated value (thousand PKR per head) of cattle and buffalo in different production systems by age-sex categories. All values are based on expert opinion. B = buffalo. C = cattle.**

Parameter	Category	Farming system										
		Smallholder subsistence		Smallholder market-oriented		Rural Commercial		Peri-urban		Corporate	Desert	
		B	C	B	C	B	C	B	C	C	B	C
Female calves <1y	min	10.5	9.0	10.5	9. 0	17.5	12.2	17.5	16.5	22.5	10.5	9.0
	max	17.5	10.0	17.5	10.0	30.0	19.4	30. 0	23.5	33. 0	17.5	10.0
Male calves <1y	min	12.5	8.0	12.5	8.0	15.5	9.8	12.5	10.0	13.5	12.5	8.0
	max	22.5	9.0	22.5	9.0	260.0	15.6	20. 0	17.5	22.5	22.5	9.0
Males >1y	min	40.0	90.0	40.0	90.0	47.5	50.0	35. 0	50.0	57.8	40.0	90.0
	max	100.0	180.0	100.0	180.0	110.0	95.0	82.5	100.0	110.0	100.0	180.0
Heifer 1-2y	min	65.0	95.0	65.0	95.0	70. 0	95.0	60. 0	110.0	115.0	65.0	95.0
	max	85.0	160.0	85.0	160.0	90. 0	160.0	90.0	175.0	182.5	85.0	160.0
Heifer 2-5y	min	85.0	70.0	85.0	70.0	100.0	117.5	90. 0	125.0	130.0	85.0	70.0
	max	170.0	90.0	170.0	90.0	195.0	165.0	180.0	185.0	200.0	170.0	90.0
Cows	min	110.0	130.0	110.0	130.0	115.0	160.0	170.0	175.0	180.0	110.0	130.0
	max	225.0	230.0	225.0	230.0	205.0	275.0	292.5	292.5	325.0	225.0	230.0

Table A6. **Proportion of value for distress sales based on that reported by** (FAO, 2016)**. The same values were used for males and females for each production system**

Age category	Category	Buffalo	Cattle
Youngstock	Min	0.39	0.39
	Max	0.47	0.47
Adults	Min	0.5	0.5
	Max	0.6	0.6

Appendix A7. **Data used for model parameters with regards to milk production. B = buffalo. C = cattle.**

Parameter	Category	Farming system											Data source
		Smallholder subsistence		Smallholder market-oriented		Rural Commercial		Peri-urban		Corporate	Desert		
		B	C	B	C	B	C	B	C	C	B	C	
Proportion of cows lactating	min	0.4	0.4	0.4	0.4	0.58	0.55	0.48	0.04	0.55	0.4	0.4	Expert opinion
	max	0.5	0.5	0.5	0.5	0.75	0.65	0.6	0.04	0.65	0.5	0.5	
Daily milk yield - non-cases	min									20	2.4	2.35	Expert opinion
	mode									25			
	max									35	4	4	
	mean	7.17	6.6	7.17	6.6	8.91	9.09	9.95	12.56				Ferrari et al., 2013
	sd	3.7	3.2	3.7	3.2	2.82	3.22	2.25	3.03				
Daily milk yield - cases (disease phase)	min									6.2	0.7	0.75	Expert opinion; Farooq et al, 2009
	mode									7.8			
	max									10.9	1.3	1.27	
	mean	1.84	2.1	1.84	2.1	2.52	2.14	2.67	3.9				Ferrari et al., 2013
	sd	1.73	2.1	1.73	2.1	1.65	1.51	1.78	1.88				
Daily milk yield - cases (recovery phase)	min									11.7	1.5	1.53	Expert opinion
	mode									14.6			
	max									20.4	2.6	2.61	
	mean	3.97	4.3	3.97	4.3	4.47	4.25	4.73	7.32				Ferrari et al., 2013
	sd	2.17	2.6	2.17	2.6	2.56	2.47	1.92	3.12				
Duration of milk drop (days, disease phase)	min	7	7	7	7	7	7	7	7	7	7	7	Ferrari et al., 2013 and author assumption
	max	10	10	10	10	10	10	10	10	10	10	10	
Duration of milk drop (days, recovery phase)	min	10	10	10	10	10	10	10	10	10	10	10	Ferrari et al., 2013 and author assumption
	mode	30	30	30	30	30	30	30	30	30	30	30	
	max	50	50	50	50	50	50	50	50	50	50	50	
Milk price per litre	min	80	65	80	65	80	65	80	65	65	80	65	Expert opinion
	mode	125	95	125	95	125	95	125	95	95	125	95	
	max	145	115	145	115	145	115	145	115	115	145	115	

Appendix A8. **Data used for treatment costs of cattle and buffalo (PKR) stratified by age-sex category. B = buffalo. C = cattle.**

Age-sex category	Category	Farming system											Data source
		Smallholder subsistence		Smallholder market-oriented		Rural Commercial		Peri-urban		Corporate	Desert		
		B	C	B	C	B	C	B	C	C	B	C	
<1 year	Min	2500	2500	2500	3000	3000	3000	4000	4000	2000	2500	2500	Expert opinion
	Mode	3500	3500	3500	3500	3500	3500	5000	5000	2500	3500	3500	
	Max	4500	4500	4500	4000	4000	4000	5000	5000	3000	4500	4500	
Heifers and males >1y	Min	3500	3500	3500	3500	3500	3500	4000	4000	3000	3500	3500	Expert opinion
	Mode	5000	5000	5000	4000	4000	4000	5000	5000	3000	5000	5000	
	Max	5500	5500	5500	4500	4500	4500	5000	5000	3000	5500	5500	
Cows	Mean	1087	1087	1087	1087	1087	850	2229	1204	1204	1087	1087	(Shah et al., 2014)
	sd	992	992	992	992	992	405	1753	394	394	992	992	

Beef feedlots

Table A9. **Parameters and data used in the beef feedlot model, based on expert opinion from 5 feedlot farms in Punjab province, Pakistan. The figures represent the combination of expert opinions with equal weighting given to each farmer. The duration of reduced feed intakes was assumed by the author. All costs in PKR.**

**Parameter**	**Symbol**	**Number of cattle on farm (probability)**		
Herd size	*hs*	30	75	200
FMD incidence	*inc*	0.32	0.38	0.44
Case fatality rate	*cfr*	0.365	0.0448	0.0563
Treatment cost (medicines per FMD case)	*tx*	1984	2497	3010
Labour cost (per minute)	*labour*	1.85	2.32	2.78
Treatment time (minutes per FMD case)	*tx_time*	23.6	34.9	42.5
Finished animal value	*fvalue*	61356	61968	62554
Finished animal value (after FMD)	$fvalue_c$	43461	44885	46418
Proportion of animal value as distress sale	$fvalue_{ds}$	0.5	-	0.75
Extended finishing time (days for FMD case)	*eft*	19.4	32.1	44.9
Normal feed intakes (kg/day DM)	di	6.3	6.5	6.7
FMD case feed intakes (kg/day DM)	di_c	0.904	1.21	1.51
Feed cost (kg DM)	fc	33.8	35.6	37.5
Duration of reduced intakes for FMD case (days)	dfmd	3	-	10
