# Supplementary material for: Economic Considerations for Advancement Through the Progressive Control Pathway: Cost–Benefit Analysis of an FMD Disease-Free Zone in Punjab Province, Pakistan
Source: Front Vet Sci. 2021 Aug 18;8:703473. doi: 10.3389/fvets.2021.703473 (PMC8416473; doi:10.3389/fvets.2021.703473)
Supplement: Supplementary Material A — Production system data. [file Data_Sheet_1.zip › Supplementary material B - Number of Outbreaks_updated.pdf]

Supplementary material B - Number of outbreaks in proposed zone and buffer areas

To estimate the overall benefits of the zone, the number of outbreaks that would be expected to occur with no control measures in place were estimated which forms the baseline scenario for comparison with the intervention. Since the vaccination campaign was initiated in 2018, the number of outbreaks for this baseline scenario was based on the typical range in the preceding years and included in the model as a stochastic parameter. For each year of the analysis, the benefits were estimated as the losses averted from outbreaks not occurring minus the cost of outbreaks that occur. This included the impacts of production and treatment of clinical cases. In year 0, this is based on the range in the actual number of outbreaks reported between 2012 and 2017 by production system, the years for which such data are available prior to implementing the mass vaccination programme in 2018 (Table B1).

To represent the anticipated benefits in the buffer zone, where control measures will also be applied, less reliable data are available on historical outbreaks. Between 2012 and 2019, between 5 and 114 outbreaks were reported annually with a median of 33.5. Based on available data, these were assumed to be divided proportionately between smallholder subsistence (14%), smallholder market-oriented (43%), rural commercial (30%) and beef feedlots (14%) farming systems. Therefore, to estimate the number of outbreaks in this zone, the overall range and median is divided proportionately among these systems (Table B2).

To account for underreporting, expert opinion was used. Prior to the implementation of mass vaccination, for all systems except the corporate and desert livestock farms it was estimated that 50-60% of outbreaks were reported rising to 90-100% after implementation of mass vaccination when farmer awareness was increased. Since there were only two corporate farms in the entire zone and buffer area, these were considered as individual farms in the model with equal chances of having an outbreak each year with all outbreaks reported. For the desert livestock farms, these are high in number (over 19,000) with typically 1-2 outbreaks reported each year. However, based on the results of NSP seroprevalence studies undertaken in 2013, the individual animal seroprevalence in animals up to 18 months of age was 55-62% with all farms sampled having at least one animal exposed. Although this study was based on convenience sampling so expected to be biased, it does provide evidence of severe under-reporting among desert livestock farms. It is for this reason that further expert opinion was gained (Appendix F). However, this also provided a large range in estimates and so therefore a large range was included in the model to account for this uncertainty which was the mean of the expert opinions (Table B1).

For expected future outbreaks, this was estimated based on the objectives of the national control strategy which was to achieve PCP stage 4 (no virus circulation) after years. During this time, a linear decline in the number of outbreaks was predicted.

Table B1. **Reported number of outbreaks by production system in the proposed zone. a) Prior to implementation of the mass vaccination campaign in 2018; b) the number of outbreaks reported in the previous 12 months at the time of the analysis; c) predicted future outbreaks each year based on a linear decline to achieve PCP stage 4 after a 5-year period as a proportion of outbreak reported in the previous 12 months (year 1 = 75%, year 2 = 50%, year 3 = 25%, year 4 = 10%, year 5 = 0%). Desert livestock farm reports based on expert opinion**

Farming system	Number of outbreaks (reported)				Number of outbreaks (predicted)				
	Pre-mass vaccination			Previous 12 months	1	2	3	4	5
	Min	Most likely	Max						
Smallholder subsistence	0	0	0	0	0	0	0	0	0
Smallholder market-oriented	10	13	20	3	3	2	1	0	0
Rural Commercial	15	21	32	1	1	0	0	0	0
Peri-Urban	1	2	4	1	1	0	0	0	0
Corporate	0	1	2	0	0	0	0	0	0
Beef feedlots	2	4	6	1	1	0	0	0	0
Desert	140	358	983	2	269	179	90	36	0

Table B2. **Estimated number of outbreaks by production system in the buffer area. These are based on the range of outbreaks reported each year and divided proportionately by production system. As for the outbreaks in the zone in Table, the predicted future outbreaks is based on a linear decline to zero outbreaks after a 5 year period.**

Farming system	Number of outbreaks (reported)				Number of outbreaks (predicted)				
	Pre-mass vaccination			Previous 12 months	1	2	3	4	5
	Min	Most likely	Max						
Smallholder subsistence	1	5	15	15	11	8	4	2	0
Smallholder market-oriented	2	14	49	49	37	25	12	5	0
Rural Commercial	1	10	34	34	26	17	9	3	0
Peri-Urban*	-	-	-	-	-	-	-	-	-
Corporate*	-	-	-	-	-	-	-	-	-
Beef feedlots	1	5	15	15	11	8	4	2	0
Desert*	-	-	-	-	-	-	-	-	-

*Not present in buffer zone
