# Supplementary material for: Economic Considerations for Advancement Through the Progressive Control Pathway: Cost–Benefit Analysis of an FMD Disease-Free Zone in Punjab Province, Pakistan
Source: Front Vet Sci. 2021 Aug 18;8:703473. doi: 10.3389/fvets.2021.703473 (PMC8416473; doi:10.3389/fvets.2021.703473)
Supplement: Supplementary Material A — Production system data. [file Data_Sheet_1.zip › Supplementary material C - Programme cost data_updated.pdf]

Supplementary material C – Programme cost data

Table C1. Model parameters and estimates used for quantifying the cost of **vaccination**.

Parameter	Value (all costs In PKR)	Parameter type in model	Information source
Vaccine (aqueous adjuvanted)	140/dose (makes up 80% of total vaccine used)	Fixed	FAO-PK
Vaccine (oil adjuvanted)	180/dose (makes up 20% of total vaccine used)	Fixed	FAO-PK
Delivery	5-10 PKR/dose	Uniform distribution	
Number of doses required (cattle - zone)	2,929,108	Fixed	FAO-PK (data from previous campaign)
Number of doses required (buffalo - zone)	1,515,431	Fixed	FAO-PK (data from previous campaign)
Number of doses required (cattle – buffer area)	615,962	Fixed	FAO-PK (data from previous campaign)
Number of doses required (buffalo – buffer area)	470,328	Fixed	FAO-PK (data from previous campaign)
% of population as youngstock	41.6%	Fixed	Pakistan Bureau of Statistics, 2006

Table C2. Model parameters and estimates used for quantifying the cost of **post-vaccination monitoring**.

Parameter	Value (all costs In PKR)	Parameter type in model	Information source
Small-scale immunogenicity studies	2.1-4.1 million PKR	Uniform distribution	Expert opinion (follow up consultation with FAO-PK)
Number of samples tested by SP ELISA for population immunogenicity study	1,050 (350 per serotype)	Fixed	FAO-OIE PVM Guidelines (Ferrari et al., 2016)
Structural protein antibody costs (per sample)	1500-2000 (most likely 1700)	Betapert	Expert opinion (staff from national laboratory)

Table C3. Model parameters and estimates used for quantifying the cost of **passive surveillance.**

Parameter	Value (all costs In PKR)	Parameter type in model	Information source
DSA for staff attending outbreaks	3000-4000 (most likely 3500) per staff member	Betapert	Expert opinion
Travel costs to attend outbreaks	2000-3000 (most likely 2500)	Betapert	Expert opinion
Antigen testing costs	2000-3000 (most likely 2500)	Betapert	Expert opinion (staff from national laboratory)
Shipping costs to national lab	500-1500 (most likely 1000)	Betapert	Expert opinion
Shipping costs to OIE reference lab (annual)	350,000-475,000	Uniform	Expert opinion (FAO-PK)

Table C4. Model parameters and estimates used for quantifying the cost of **active surveillance.**

Parameter	Value (all costs In PKR)	Parameter type in model	Information source
Sample size for NSP seroprevalence studies (50% seroprevalence in year 1)	385 farms, 9000 samples	Fixed	(Thrusfield et al., 2001)
Sample size for NSP seroprevalence studies (40% seroprevalence in year 2)	369 farms, 8500 samples	Fixed	(Thrusfield et al., 2001)
Sample size for NSP seroprevalence studies (30% seroprevalence in year 3)	323 farms, 7500 samples	Fixed	(Thrusfield et al., 2001)
Sample size for NSP seroprevalence studies (20% seroprevalence in year 4)	246 farms, 5700 samples	Fixed	(Thrusfield et al., 2001)
Sample size for NSP seroprevalence studies (10% seroprevalence in year 5)	139 farms, 3200 samples	Fixed	(Thrusfield et al., 2001)
Sample size for NSP seroprevalence studies (when free)	300 farms, 7000 samples	Fixed	(Thrusfield et al., 2001)

Number of farms visited per day for serosurvey	2-8 (most likely 4.5)	Betapert	Assumed by authors
Number of full-time veterinary officers	15-25	Uniform	Assumed by authors
Salary for field veterinary officer and assistant managers	75,000 PKR/month	Fixed	FAO-PK

Table C5. Model parameters and estimates used for quantifying the cost of **sanitary measures.**

Parameter	Value (all costs In PKR)	Parameter type in model	Information source
Quarantine stations at border with Sindh province	3-10 (most likely 5)	Betapert	Assumed by authors
Initial consultancy costs for establishing quarantine station (year 1 only)	4.95m	Fixed	Assumed by authors
Cost of building quarantine station (year 2)	4.2m-4.95m each	Uniform	Assumed by authors
Maintenance costs of quarantine station (year 3 onwards)	250,000-495,000 each per year	Uniform	Assumed by authors

Table C6. Model parameters and estimates used for quantifying the cost of **Programme management.**

Parameter	Value (all costs In PKR)	Parameter type in model	Information source
Salary for programme manager (every year)	150,000 per month	Fixed	Assumed by authors
Initial costs to establish public private partnership (year 1 only)	1.04m-1.85m per year	Uniform	Assumed by authors
Ongoing costs to support public private partnership (year 2 onwards)	1.58m-3.0m per year	Uniform	Assumed by authors
Stakeholder communication costs (year 1 and 2 only)	3.5m-5.25m per year	Uniform	Assumed by authors

Stakeholder communication costs (year 3 onwards)	2.0m-3.0m per year	Unform	Assumed by authors

Table C7. Model parameters and estimates used for quantifying the cost of **Preparatory studies, training and capacity building.**

Parameter	Value (all costs In PKR)	Parameter type in model	Information source
Risk assessment and value chain analysis – cost of consultants and to train staff in Pakistan (initial studies in year 1 only)	3.85m-5.99m	Uniform	Assumed by authors
Yearly updates to risk assessment and value chain analysis (year 2 onwards)	625,000-1.065m per year	Uniform	Assumed by authors
Refresher training in risk assessment and value chain analysis (every 3 years)	1.114m-1.8m	Uniform	Assumed by authors
Surveillance and PVM strategy development (workshops and training – year 1 only)	4.25m-7.5m	Uniform	Assumed by authors
Research grant to establish the role of small ruminants in FMD epidemiology (year 1 only)	6.25m-10.375m	Uniform	Assumed by authors
Training in outbreak investigation (initial training in year 1 only)	5.623-6.875	Uniform	Assumed by authors
Training in outbreak investigation (refresher every 3 years)	1.875m-3.5m	Uniform	Assumed by authors
Laboratory training in VNT and vaccine matching	1.5m-3.0m	Uniform	Assumed by authors
Establish QA system in laboratory	1.5m-3.0m	Uniform	Assumed by authors

Refresher training in laboratory (every 3 years)	850,000-1.5m	Uniform	Assumed by authors
Participate in proficiency tests (every year)	400,000-800,000 per year	Uniform	Assumed by authors
Development of preparedness and contingency plans (years 2 and 3)	1.875m-3.75m	Uniform	Assumed by authors
Building export abattoir in zone (year 2 only)	671.01m	Fixed	Assumed by authors
