# Supplementary material for: Economic Considerations for Advancement Through the Progressive Control Pathway: Cost–Benefit Analysis of an FMD Disease-Free Zone in Punjab Province, Pakistan
Source: Front Vet Sci. 2021 Aug 18;8:703473. doi: 10.3389/fvets.2021.703473 (PMC8416473; doi:10.3389/fvets.2021.703473)
Supplement: Supplementary Material A — Production system data. [file Data_Sheet_1.zip › Supplementary material D - Expert opinion exercise for workshop_updated.pdf]

Supplementary material D

FMD cost model –
Further data needed and assumptions to query

1. Baseline production

Number of farms

1. *Estimate the **number of farms** present in each **province** by **production system**. Please state the range*

Province	Production system	Minimum	Maximum	Most likely	Confidence*(1-5)
Azad Jammu and Kashmir	Smallholder subsistence				
	Smallholder market-orientated				
	Rural commercial				
	Peri-Urban				
	Corporate				
	Dairy colonies				
Balochistan	Smallholder subsistence				
	Smallholder market-orientated				
	Rural commercial				
	Peri-Urban				
	Corporate				
	Dairy colonies				
Gilgit-Baltistan	Smallholder subsistence				
	Smallholder market-orientated				
	Rural commercial				
	Peri-Urban				
	Corporate				
	Dairy colonies				
Islamabad Capital Territory	Smallholder subsistence				
	Smallholder market-orientated				
	Rural commercial				
	Peri-Urban				
	Corporate				
	Dairy colonies				
Khyber Pakhtunkhwa	Smallholder subsistence				
	Smallholder market-orientated				
	Rural commercial				
	Peri-Urban				
	Corporate				
	Dairy colonies				
Punjab	Smallholder subsistence				
	Smallholder market-orientated				
	Rural commercial				
	Peri-Urban				
	Corporate				
	Dairy colonies				
Sindh	Smallholder subsistence				
	Smallholder market-orientated				
	Rural commercial				
	Peri-Urban				
	Corporate				
	Dairy colonies				

Fertility

1. *Below are the **fertility parameters** used in the economic model for the different production systems. If you disagree with them, indicate the parameters you think are more reasonable based on your experience. The definitions of the terms are provided.*

Parameter	Production system	Min	Max	New min	New max	Confidence (1-5)
**Pregnancy/conception rate in COWS** (percentage of services that result in a successful offspring)	Smallholder subsistence	20%	50%			
	Smallholder market-orientated	20%	50%			
	Rural commercial	20%	50%			
	Peri-Urban	No breeding				
	Corporate	20%	50%			
	Dairy colonies	No breeding				
**Pregnancy/conception rate in HEIFERS** (percentage of services that result in a successful offspring)	Smallholder subsistence	30%	70%			
	Smallholder market-orientated	30%	70%			
	Rural commercial	30%	70%			
	Peri-Urban	No breeding				
	Corporate	30%	70%			
	Dairy colonies	No breeding				
**Heat detection rate in COWS** (percentage of oestrus events that are served – either through AI or a bull)	Smallholder subsistence	20%	60%			
	Smallholder market-orientated	20%	60%			
	Rural commercial	20%	60%			
	Peri-Urban	No breeding				
	Corporate	40%	70%			
	Dairy colonies	No breeding				
**Heat detection rate in HEIFERS** (percentage of oestrus events that are served – either through AI or a bull)	Smallholder subsistence	30%		70%		
	Smallholder market-orientated	30%		70%		
	Rural commercial	30%		70%		
	Peri-Urban	No breeding				
	Corporate	50%		80%		
	Dairy colonies	No breeding				
**Voluntary waiting period** (number of days after calving that farms will **start** breeding cows)	Smallholder subsistence	60 days		150 days		
	Smallholder market-orientated	60 days		150 days		
	Rural commercial	60 days		150 days		
	Peri-Urban	No breeding				
	Corporate	60 days		80 days		
	Dairy colonies	No breeding				

Mortality rates

1. *Please estimate the **baseline mortality estimates** for the different animal types by production system. Please give the **annual % expected** mortality.*

System	Animal category	Minimum %	Maximum %	Most likely %	Confidence* (1-5)
Smallholder subsistence	Male calves (<1 year old)				
	Female calves (<1 year old)				
	Bulls (>1 year old)				
	Heifers (>1 year old)				
	Cows				
Smallholder market-orientated	Male calves (<1 year old)				
	Female calves (<1 year old)				
	Bulls (>1 year old)				
	Heifers (>1 year old)				
	Cows				
Rural commercial	Male calves (<1 year old)				
	Female calves (<1 year old)				
	Bulls (>1 year old)				
	Heifers (>1 year old)				
	Cows				
Peri-Urban	Male calves (<1 year old)				
	Female calves (<1 year old)				
	Bulls (>1 year old)				
	Heifers (>1 year old)				
	Cows				
Corporate	Male calves (<1 year old)				
	Female calves (<1 year old)				
	Bulls (>1 year old)				
	Heifers (>1 year old)				
	Cows				
Dairy colonies	Male calves (<1 year old)				
	Female calves (<1 year old)				
	Bulls (>1 year old)				
	Heifers (>1 year old)				
	Cows				

Offtake rates

1. *Please estimate the* ***baseline offtake rates*** *for the different animal types by production system. Please give the annual % of animals that will be sold (this could be due to culling or to generate revenue as part of normal farm policy).*

System	Animal category	Minimum %	Maximum %	Most likely %	Confidence*(1-5)
Smallholder subsistence	Male calves (<1 year old)				
	Female calves (<1 year old)				
	Bulls (>1 year old)				
	Heifers (>1 year old)				
	Cows				
Smallholder market-orientated	Male calves (<1 year old)				
	Female calves (<1 year old)				
	Bulls (>1 year old)				
	Heifers (>1 year old)				
	Cows				
Rural commercial	Male calves (<1 year old)				
	Female calves (<1 year old)				
	Bulls (>1 year old)				
	Heifers (>1 year old)				
	Cows				
Peri-Urban	Male calves (<1 year old)				
	Female calves (<1 year old)				
	Bulls (>1 year old)				
	Heifers (>1 year old)				
	Cows				
Corporate	Male calves (<1 year old)				
	Female calves (<1 year old)				
	Bulls (>1 year old)				
	Heifers (>1 year old)				
	Cows				
Dairy colonies	Male calves (<1 year old)				
	Female calves (<1 year old)				
	Bulls (>1 year old)				
	Heifers (>1 year old)				
	Cows				

Animal prices

1. *Below are the* ***estimated livestock prices*** *used in the economic model for the different production systems. If you disagree with them, indicate the parameters you think are more reasonable based on your experience. The definitions of the terms are provided.*

Animal prices - <u>BUFFALO</u>

Parameter	Production system	Min	Max	New min	New max	Confidence (1-5)
Female calf (<1yo)	Smallholder subsistence	10,000	15000			
	Smallholder market-orientated	10,000	15000			
	Rural commercial	10,000	15000			
	Peri-Urban	10,000	15000			
	Corporate	No buffalo				
	Dairy colonies	10,000	15000			
Male calf (<1yo)	Smallholder subsistence	10,000	15000			
	Smallholder market-orientated	10,000	15000			
	Rural commercial	10,000	15000			
	Peri-Urban	10,000	15000			
	Corporate	No buffalo				
	Dairy colonies	10,000	15000			
Male (>1yo)	Smallholder subsistence	75,000	80,000			
	Smallholder market-orientated	75,000	80,000			
	Rural commercial	75,000	80,000			
	Peri-Urban	75,000	80,000			
	Corporate	No buffalo				
	Dairy colonies	75,000	80,000			
Heifer (1-2yo)	Smallholder subsistence	75,000	80,000			
	Smallholder market-orientated	75,000	80,000			
	Rural commercial	75,000	80,000			
	Peri-Urban	75,000	80,000			
	Corporate	No buffalo				
	Dairy colonies	75,000	80,000			
Heifer (2-3yo)	Smallholder subsistence	75,000	80,000			
	Smallholder market-orientated	75,000	80,000			
	Rural commercial	75,000	80,000			
	Peri-Urban	75,000	80,000			
	Corporate	No buffalo				
	Dairy colonies	75,000	80,000			
Heifer (3-4yo)	Smallholder subsistence	75,000	80,000			
	Smallholder market-orientated	75,000	80,000			
	Rural commercial	75,000	80,000			
	Peri-Urban	75,000	80,000			
	Corporate	No buffalo				
	Dairy colonies	75,000	80,000			

Heifer (4-5yo)	Smallholder subsistence	75,000	80,000			
	Smallholder market-orientated	75,000	80,000			
	Rural commercial	75,000	80,000			
	Peri-Urban	75,000	80,000			
	Corporate	No buffalo				
	Dairy colonies	75,000	80,000			
Cow	Smallholder subsistence	140,000	215,000			
	Smallholder market-orientated	140,000	215,000			
	Rural commercial	140,000	215,000			
	Peri-Urban	140,000	215,000			
	Corporate	No buffalo				
	Dairy colonies	140,000	215,000			

Animal prices - __CATTLE__

Parameter	Production system	Min	Max	New min	New max	Confidence (1-5)
Female calf (<1yo)	Smallholder subsistence	No cattle				
	Smallholder market-orientated	No cattle				
	Rural commercial	10,000	15000			
	Peri-Urban	10,000	15000			
	Corporate	10,000	15000			
	Dairy colonies	10,000	15000			
Male calf (<1yo)	Smallholder subsistence	No cattle				
	Smallholder market-orientated	No cattle				
	Rural commercial	10,000	15000			
	Peri-Urban	10,000	15000			
	Corporate	10,000	15000			
	Dairy colonies	10,000	15000			
Male (>1yo)	Smallholder subsistence	No cattle				
	Smallholder market-orientated	No cattle				
	Rural commercial	75,000	80,000			
	Peri-Urban	75,000	80,000			
	Corporate	75,000	80,000			
	Dairy colonies	75,000	80,000			
Heifer (1-2yo)	Smallholder subsistence	No cattle				
	Smallholder market-orientated	No cattle				
	Rural commercial	75,000	80,000			
	Peri-Urban	75,000	80,000			
	Corporate	75,000	80,000			
	Dairy colonies	75,000	80,000			
Heifer (2-3yo)	Smallholder subsistence	No cattle				
	Smallholder market-orientated	No cattle				
	Rural commercial	75,000	80,000			
	Peri-Urban	75,000	80,000			
	Corporate	75,000	80,000			
	Dairy colonies	75,000	80,000			
Heifer (3-4yo)	Smallholder subsistence	No cattle				
	Smallholder market-orientated	No cattle				
	Rural commercial	75,000	80,000			
	Peri-Urban	75,000	80,000			
	Corporate	75,000	80,000			
	Dairy colonies	75,000	80,000			
Heifer (4-5yo)	Smallholder subsistence	No cattle				
	Smallholder market-orientated	No cattle				
	Rural commercial	75,000	80,000			
	Peri-Urban	75,000	80,000			
	Corporate	75,000	80,000			
	Dairy colonies	75,000	80,000			

Cow	Smallholder subsistence	No cattle				
	Smallholder market-orientated	No cattle				
	Rural commercial	140,000	215,000			
	Peri-Urban	140,000	215,000			
	Corporate	140,000	215,000			
	Dairy colonies	140,000	215,000			

Lactating cows

1. *Among the different production systems, what is the range in the* ***percentage of cows are lactating*** *(as opposed to dry, not including heifers)? Below are the parameters used in the model. If you disagree with them, indicate what you think and how confident you are in your estimate.*

Species	Production system	Min %	Max %	New min %	New max %	Confidence (1-5)
Buffalo	Smallholder subsistence	40	50			
	Smallholder market-orientated	40	50			
	Rural commercial	50	60			
	Peri-Urban	90	100			
	Corporate	No buffalo				
	Dairy colonies	90	100			
Cattle	Smallholder subsistence	No cattle				
	Smallholder market-orientated	No cattle				
	Rural commercial	50	60			
	Peri-Urban	90	100			
	Corporate	60	70			
	Dairy colonies	90	100			

Milk yields

1. *Among the different production systems, what is a typical* ***milk yield*** *(litres/cow/day)?*

Species	Production system	Min (litres/cow/day)	Max (litres/cow/day)	Most likely (litres/cow/day)	Confidence (1-5)
Buffalo	Smallholder subsistence				
	Smallholder market-orientated				
	Rural commercial				
	Peri-Urban				
	Corporate	No buffalo			
	Dairy colonies				
Cattle	Smallholder subsistence	No cattle			
	Smallholder market-orientated	No cattle			
	Rural commercial				
	Peri-Urban				
	Corporate				
	Dairy colonies				

Milk price

1. *What is the milk price for milk sold in different production systems?*

Species	Production system	Min (PKR/litre)	Max (PKR/litre)	Most likely (PKR/litre)	Confidence (1-5)
Buffalo	Smallholder subsistence	No milk sold			
	Smallholder market-orientated				
	Rural commercial				
	Peri-Urban				
	Corporate	No buffalo			
	Dairy colonies				
Cattle	Smallholder subsistence	No cattle			
	Smallholder market-orientated	No cattle			
	Rural commercial				
	Peri-Urban				
	Corporate				
	Dairy colonies				

Draught power

1. *What percentage of farms use **draught power**?*

Province	Production system	Minimum	Maximum	Most likely	Confidence*(1-5)
Azad Jammu and Kashmir	Smallholder subsistence				
	Smallholder market-orientated				
	Rural commercial				
	Peri-Urban				
	Corporate				
	Dairy colonies				
Balochistan	Smallholder subsistence				
	Smallholder market-orientated				
	Rural commercial				
	Peri-Urban				
	Corporate				
	Dairy colonies				
Gilgit-Baltistan	Smallholder subsistence				
	Smallholder market-orientated				
	Rural commercial				
	Peri-Urban				
	Corporate				
	Dairy colonies				
Islamabad Capital Territory	Smallholder subsistence				
	Smallholder market-orientated				
	Rural commercial				
	Peri-Urban				
	Corporate				
	Dairy colonies				
Khyber Pakhtunkhwa	Smallholder subsistence				
	Smallholder market-orientated				
	Rural commercial				
	Peri-Urban				
	Corporate				
	Dairy colonies				
Punjab	Smallholder subsistence				
	Smallholder market-orientated				
	Rural commercial				
	Peri-Urban				
	Corporate				
	Dairy colonies				
Sindh	Smallholder subsistence				
	Smallholder market-orientated				
	Rural commercial				
	Peri-Urban				
	Corporate				
	Dairy colonies				

2. FMD Incidence

Reported outbreaks

1. *Estimate the **number of <u>reported</u> outbreaks** per year by production system in each province*

Province	Production system	Minimum	Maximum	Most likely	Confidence*(1-5)
Azad Jammu and Kashmir	Smallholder subsistence				
	Smallholder market-orientated				
	Rural commercial				
	Peri-Urban				
	Corporate				
	Dairy colonies				
Balochistan	Smallholder subsistence				
	Smallholder market-orientated				
	Rural commercial				
	Peri-Urban				
	Corporate				
	Dairy colonies				
Gilgit-Baltistan	Smallholder subsistence				
	Smallholder market-orientated				
	Rural commercial				
	Peri-Urban				
	Corporate				
	Dairy colonies				
Islamabad Capital Territory	Smallholder subsistence				
	Smallholder market-orientated				
	Rural commercial				
	Peri-Urban				
	Corporate				
	Dairy colonies				
Khyber Pakhtunkhwa	Smallholder subsistence				
	Smallholder market-orientated				
	Rural commercial				
	Peri-Urban				
	Corporate				
	Dairy colonies				
Punjab	Smallholder subsistence				
	Smallholder market-orientated				
	Rural commercial				
	Peri-Urban				
	Corporate				
	Dairy colonies				
Sindh	Smallholder subsistence				
	Smallholder market-orientated				
	Rural commercial				
	Peri-Urban				
	Corporate				
	Dairy colonies				

Confidence scoring (1-5) with 1 = not confident and 5 = very confident

Outbreak reporting

2. *What **percentage** of all outbreaks are actually **reported**? Please give your estimates by province and production system.*

Province	Production system	Minimum	Maximum	Most likely	Confidence*(1-5)
Azad Jammu and Kashmir	Smallholder subsistence				
	Smallholder market-orientated				
	Rural commercial				
	Peri-Urban				
	Corporate				
	Dairy colonies				
Balochistan	Smallholder subsistence				
	Smallholder market-orientated				
	Rural commercial				
	Peri-Urban				
	Corporate				
	Dairy colonies				
Gilgit-Baltistan	Smallholder subsistence				
	Smallholder market-orientated				
	Rural commercial				
	Peri-Urban				
	Corporate				
	Dairy colonies				
Islamabad Capital Territory	Smallholder subsistence				
	Smallholder market-orientated				
	Rural commercial				
	Peri-Urban				
	Corporate				
	Dairy colonies				
Khyber Pakhtunkhwa	Smallholder subsistence				
	Smallholder market-orientated				
	Rural commercial				
	Peri-Urban				
	Corporate				
	Dairy colonies				
Punjab	Smallholder subsistence				
	Smallholder market-orientated				
	Rural commercial				
	Peri-Urban				
	Corporate				
	Dairy colonies				
Sindh	Smallholder subsistence				
	Smallholder market-orientated				
	Rural commercial				
	Peri-Urban				
	Corporate				
	Dairy colonies				

FMD case incidence

1. *The incidence by age presented by Ferrari et al (2013) is used in the model. Please indicate if you think this is varies by different production systems in the table below.*

System	Animal category	Incidence %	Maximum %	Most likely %	Confidence*(1-5)
Smallholder subsistence	Male calves (<1 year old)				
	Female calves (<1 year old)				
	Bulls (>1 year old)				
	Heifers (>1 year old)				
	Cows				
Smallholder market-orientated	Male calves (<1 year old)				
	Female calves (<1 year old)				
	Bulls (>1 year old)				
	Heifers (>1 year old)				
	Cows				
Rural commercial	Male calves (<1 year old)				
	Female calves (<1 year old)				
	Bulls (>1 year old)				
	Heifers (>1 year old)				
	Cows				
Peri-Urban	Male calves (<1 year old)				
	Female calves (<1 year old)				
	Bulls (>1 year old)				
	Heifers (>1 year old)				
	Cows				
Corporate	Male calves (<1 year old)				
	Female calves (<1 year old)				
	Bulls (>1 year old)				
	Heifers (>1 year old)				
	Cows				
Dairy colonies	Male calves (<1 year old)				
	Female calves (<1 year old)				
	Bulls (>1 year old)				
	Heifers (>1 year old)				
	Cows				

3. Impact of FMD cases

Distress sales

1. *What percentage of FMD cases are sold as **distress sales**? Below is the range used in the current economic model. If you disagree, please insert the new numbers in the columns provided.*

Distress sales - BUFFALO

**System**	**Animal category**	**Min %**	**Max %**	**New min (%)**	**New max (%)**	**Confidence* (1-5)**
Smallholder subsistence	Male calves (<1 year old)	0	10			
	Female calves (<1 year old)	0	10			
	Bulls (>1 year old)	0	10			
	Heifers (>1 year old)	0	10			
	Cows	0	10			
Smallholder market-orientated	Male calves (<1 year old)	0	10			
	Female calves (<1 year old)	0	10			
	Bulls (>1 year old)	0	10			
	Heifers (>1 year old)	0	10			
	Cows	0	10			
Rural commercial	Male calves (<1 year old)	0	10			
	Female calves (<1 year old)	0	10			
	Bulls (>1 year old)	0	10			
	Heifers (>1 year old)	0	10			
	Cows	0	10			
Peri-Urban	Male calves (<1 year old)	0	20			
	Female calves (<1 year old)	0	20			
	Bulls (>1 year old)	0	20			
	Heifers (>1 year old)	0	20			
	Cows	0	20			
Corporate	Male calves (<1 year old)	0	5			
	Female calves (<1 year old)	0	5			
	Bulls (>1 year old)	0	5			
	Heifers (>1 year old)	0	5			
	Cows	0	5			
Dairy colonies	Male calves (<1 year old)	0	20			
	Female calves (<1 year old)	0	20			
	Bulls (>1 year old)	0	20			
	Heifers (>1 year old)	0	20			
	Cows	0	20			

Distress sales - CATTLE

System	Animal category	Min %	Max %	New min (%)	New max (%)	Confidence* (1-5)
Smallholder subsistence	Male calves (<1 year old)	0	10			
	Female calves (<1 year old)	0	10			
	Bulls (>1 year old)	0	10			
	Heifers (>1 year old)	0	10			
	Cows	0	10			
Smallholder market-orientated	Male calves (<1 year old)	0	10			
	Female calves (<1 year old)	0	10			
	Bulls (>1 year old)	0	10			
	Heifers (>1 year old)	0	10			
	Cows	0	10			
Rural commercial	Male calves (<1 year old)	0	10			
	Female calves (<1 year old)	0	10			
	Bulls (>1 year old)	0	10			
	Heifers (>1 year old)	0	10			
	Cows	0	10			
Peri-Urban	Male calves (<1 year old)	0	20			
	Female calves (<1 year old)	0	20			
	Bulls (>1 year old)	0	20			
	Heifers (>1 year old)	0	20			
	Cows	0	20			
Corporate	Male calves (<1 year old)	0	5			
	Female calves (<1 year old)	0	5			
	Bulls (>1 year old)	0	5			
	Heifers (>1 year old)	0	5			
	Cows	0	5			
Dairy colonies	Male calves (<1 year old)	0	20			
	Female calves (<1 year old)	0	20			
	Bulls (>1 year old)	0	20			
	Heifers (>1 year old)	0	20			
	Cows	0	20			

Culling rates of FMD cases

1. *What percentage of FMD cases are **culled** due to FMD? These are not distress sales which are sold when sick, rather end up being sold due to poor productivity. Below is the range used in the current economic model. If you disagree, please insert the new numbers in the columns provided.*

FMD culling rates - BUFFALO

**System**	**Animal category**	**Min %**	**Max %**	**New min (%)**	**New max (%)**	**Confidence* (1-5)**
Smallholder subsistence	Male calves (<1 year old)	0	0			
	Female calves (<1 year old)	0	0			
	Bulls (>1 year old)	0	0			
	Heifers (>1 year old)	0	0			
	Cows	0	5			
Smallholder market-orientated	Male calves (<1 year old)	0	0			
	Female calves (<1 year old)	0	0			
	Bulls (>1 year old)	0	0			
	Heifers (>1 year old)	0	0			
	Cows	0	10			
Rural commercial	Male calves (<1 year old)	0	0			
	Female calves (<1 year old)	0	0			
	Bulls (>1 year old)	0	0			
	Heifers (>1 year old)	0	0			
	Cows	0	15			
Peri-Urban	Male calves (<1 year old)	0	0			
	Female calves (<1 year old)	0	0			
	Bulls (>1 year old)	0	0			
	Heifers (>1 year old)	0	0			
	Cows	0	15			
Corporate	Male calves (<1 year old)	0	0			
	Female calves (<1 year old)	0	0			
	Bulls (>1 year old)	0	0			
	Heifers (>1 year old)	0	0			
	Cows	0	10			
Dairy colonies	Male calves (<1 year old)	0	0			
	Female calves (<1 year old)	0	0			
	Bulls (>1 year old)	0	0			
	Heifers (>1 year old)	0	0			
	Cows	0	15			

FMD culling rates - CATTLE

System	Animal category	Min %	Max %	New min (%)	New max (%)	Confidence* (1-5)
Smallholder subsistence	Male calves (<1 year old)	0	0			
	Female calves (<1 year old)	0	0			
	Bulls (>1 year old)	0	0			
	Heifers (>1 year old)	0	0			
	Cows	0	5			
Smallholder market-orientated	Male calves (<1 year old)	0	0			
	Female calves (<1 year old)	0	0			
	Bulls (>1 year old)	0	0			
	Heifers (>1 year old)	0	0			
	Cows	0	10			
Rural commercial	Male calves (<1 year old)	0	0			
	Female calves (<1 year old)	0	0			
	Bulls (>1 year old)	0	0			
	Heifers (>1 year old)	0	0			
	Cows	0	15			
Peri-Urban	Male calves (<1 year old)	0	0			
	Female calves (<1 year old)	0	0			
	Bulls (>1 year old)	0	0			
	Heifers (>1 year old)	0	0			
	Cows	0	15			
Corporate	Male calves (<1 year old)	0	0			
	Female calves (<1 year old)	0	0			
	Bulls (>1 year old)	0	0			
	Heifers (>1 year old)	0	0			
	Cows	0	10			
Dairy colonies	Male calves (<1 year old)	0	0			
	Female calves (<1 year old)	0	0			
	Bulls (>1 year old)	0	0			
	Heifers (>1 year old)	0	0			
	Cows	0	15			

Case fatality rates

1. *What percentage of FMD cases **die due to FMD**? These are deaths in the acute phase and not distress sales. Below is the range used in the current economic model. If you disagree, please insert the new numbers in the columns provided.*

FMD case fatality – <u>BUFFALO</u>

System	Animal category	Min %	Max %	Most likely (%)	New min (%)	New max (%)	New most likely (%)	Confidence* (1-5)
Smallholder subsistence	Male calves (<1 year old)	24	44	34				
	Female calves (<1 year old)	24	44	34				
	Bulls (>1 year old)	5	25	15				
	Heifers (>1 year old)	5	25	15				
	Cows	2.2	4.2	3.2				
Smallholder market-orientated	Male calves (<1 year old)	24	44	34				
	Female calves (<1 year old)	24	44	34				
	Bulls (>1 year old)	5	25	15				
	Heifers (>1 year old)	5	25	15				
	Cows	2.2	4.2	3.2				
Rural commercial	Male calves (<1 year old)	24	44	34				
	Female calves (<1 year old)	24	44	34				
	Bulls (>1 year old)	5	25	15				
	Heifers (>1 year old)	5	25	15				
	Cows	2.2	4.2	3.2				
Peri-Urban	Male calves (<1 year old)	24	44	34				
	Female calves (<1 year old)	24	44	34				
	Bulls (>1 year old)	5	25	15				
	Heifers (>1 year old)	5	25	15				
	Cows	2.2	4.2	3.2				
Corporate	Male calves (<1 year old)	No buffalo						
	Female calves (<1 year old)	No buffalo						
	Bulls (>1 year old)	No buffalo						
	Heifers (>1 year old)	No buffalo						
	Cows	No buffalo						
Dairy colonies	Male calves (<1 year old)	24	44	34				
	Female calves (<1 year old)	24	44	34				
	Bulls (>1 year old)	5	25	15				
	Heifers (>1 year old)	5	25	15				
	Cows	2.2	4.2	3.2				

FMD case fatality – <u>CATTLE</u>

System	Animal category	Min %	Max %	Most likely (%)	New min (%)	New max (%)	New most likely (%)	Confidence* (1-5)
Smallholder subsistence	Male calves (<1 year old)	No cattle						
	Female calves (<1 year old)	No cattle						
	Bulls (>1 year old)	No cattle						
	Heifers (>1 year old)	No cattle						
	Cows	No cattle						
Smallholder market-orientated	Male calves (<1 year old)	No cattle						
	Female calves (<1 year old)	No cattle						
	Bulls (>1 year old)	No cattle						
	Heifers (>1 year old)	No cattle						
	Cows	No cattle						
Rural commercial	Male calves (<1 year old)	21	41	31				
	Female calves (<1 year old)	21	41	31				
	Bulls (>1 year old)	10	30	20				
	Heifers (>1 year old)	10	30	20				
	Cows	3.5	5.5	4.5				
Peri-Urban	Male calves (<1 year old)	21	41	31				
	Female calves (<1 year old)	21	41	31				
	Bulls (>1 year old)	10	30	20				
	Heifers (>1 year old)	10	30	20				
	Cows	3.5	5.5	4.5				
Corporate	Male calves (<1 year old)	8	16	12				
	Female calves (<1 year old)	8	16	12				
	Bulls (>1 year old)	2	12	7				
	Heifers (>1 year old)	2	12	7				
	Cows	1	10	5				
Dairy colonies	Male calves (<1 year old)	21	41	31				
	Female calves (<1 year old)	21	41	31				
	Bulls (>1 year old)	10	30	20				
	Heifers (>1 year old)	10	30	20				
	Cows	3.5	5.5	4.5				

Abortion rates

1. *What percentage of cows and heifers **abort** due to FMD?*

Species	Animal category	Min %	Max %	Most likely (%)	New min (%)	New max (%)	New most likely (%)	Confidence* (1-5)
Buffalo	Heifers	0	10	4				
	Cows	0	10	4				
Cattle	Heifers	10	18	25				
	Cows	10	25	15				

5. Reaction and expenditure

FMD vaccination

1. *Estimate the percentage of farms that are vaccinated by province and production system. This is **preventive** vaccination (NOT emergency) using **IMPORTED** vaccines.*

Province	Production system	Minimum	Maximum	Most likely	Confidence*(1-5)
Azad Jammu and Kashmir	Smallholder subsistence				
	Smallholder market-orientated				
	Rural commercial				
	Peri-Urban				
	Corporate				
	Dairy colonies				
Balochistan	Smallholder subsistence				
	Smallholder market-orientated				
	Rural commercial				
	Peri-Urban				
	Corporate				
	Dairy colonies				
Gilgit-Baltistan	Smallholder subsistence				
	Smallholder market-orientated				
	Rural commercial				
	Peri-Urban				
	Corporate				
	Dairy colonies				
Islamabad Capital Territory	Smallholder subsistence				
	Smallholder market-orientated				
	Rural commercial				
	Peri-Urban				
	Corporate				
	Dairy colonies				
Khyber Pakhtunkhwa	Smallholder subsistence				
	Smallholder market-orientated				
	Rural commercial				
	Peri-Urban				
	Corporate				
	Dairy colonies				
Punjab	Smallholder subsistence				
	Smallholder market-orientated				
	Rural commercial				
	Peri-Urban				
	Corporate				
	Dairy colonies				
Sindh	Smallholder subsistence				
	Smallholder market-orientated				
	Rural commercial				
	Peri-Urban				
	Corporate				
	Dairy colonies				

2. *Estimate the percentage of farms that are vaccinated by province and production system. This is **preventive** vaccination (NOT emergency) using **LOCALLY PRODUCED** vaccines.*

Province	Production system	Minimum	Maximum	Most likely	Confidence*(1-5)
Azad Jammu and Kashmir	Smallholder subsistence				
	Smallholder market-orientated				
	Rural commercial				
	Peri-Urban				
	Corporate				
	Dairy colonies				
Balochistan	Smallholder subsistence				
	Smallholder market-orientated				
	Rural commercial				
	Peri-Urban				
	Corporate				
	Dairy colonies				
Gilgit-Baltistan	Smallholder subsistence				
	Smallholder market-orientated				
	Rural commercial				
	Peri-Urban				
	Corporate				
	Dairy colonies				
Islamabad Capital Territory	Smallholder subsistence				
	Smallholder market-orientated				
	Rural commercial				
	Peri-Urban				
	Corporate				
	Dairy colonies				
Khyber Pakhtunkhwa	Smallholder subsistence				
	Smallholder market-orientated				
	Rural commercial				
	Peri-Urban				
	Corporate				
	Dairy colonies				
Punjab	Smallholder subsistence				
	Smallholder market-orientated				
	Rural commercial				
	Peri-Urban				
	Corporate				
	Dairy colonies				
Sindh	Smallholder subsistence				
	Smallholder market-orientated				
	Rural commercial				
	Peri-Urban				
	Corporate				
	Dairy colonies				

3. *Estimate the percentage of outbreaks that are **attended by veterinary staff** in each province.*

Province	Minimum	Maximum	Most likely	Confidence*(1-5)
Azad Jammu and Kashmir				
Balochistan				
Gilgit-Baltistan				
Islamabad Capital Territory				
Khyber Pakhtunkhwa				
Punjab				
Sindh				

4. *What is the cost of sending samples to the laboratory?*

Province	Fixed cost
Azad Jammu and Kashmir	
Balochistan	
Gilgit-Baltistan	
Islamabad Capital Territory	
Khyber Pakhtunkhwa	
Punjab	
Sindh	

5. *How many people typically attend an outbreak?*

Province	Fixed number
Azad Jammu and Kashmir	
Balochistan	
Gilgit-Baltistan	
Islamabad Capital Territory	
Khyber Pakhtunkhwa	
Punjab	
Sindh	

For those working in the laboratory:

6. For each epithelium sample submitted, which tests are typically done?
7. Please state the estimated cost of the following laboratory tests

Possible aspects to include (production impacts):

a) Fertility (include clear definitions)
    a. Number of cycles missed with cases of FMD
    b. Case extension to age at first calving
b) Present morbidity data (used Ferrari's data) and ask if agree, and if different in other systems
c) Treatment costs

Possible aspects to include (reaction)
a) Vaccination
    a. Delivery prices
    b. Vaccine prices
b) Sanitary measures
    a. What is done? What costs are involved?
c) Trainings conducted
    a. Number on FMD each year
    b. Number of attendees
    c. Estimated costs per province (Maybe just ask this)
    d. National level trainings
d) Communications

Specific lab questions
a) Tests routinely done on samples submitted
b) Cost of each test (also for NSP)
c) LIMS maintenance
d) Staff trainings (number each year)

Weighting the responses

1. Years of experience
2. Confidence in the results

Options:
1. Have individuals reply and weight answers according to a combination of their years of experience and the confidence in the results
2. Have group responses, and weight just by the confidence in the results. Groups can be 2-3 people (so if four large groups in total, these can be split in two)
3. As 2, but each member of the group states their years of experience which are combined for the group weighting with the confidence in the results

Give each section per group. But for regional differences will need people from the provinces.
