# Supplementary material for: Economic Considerations for Advancement Through the Progressive Control Pathway: Cost–Benefit Analysis of an FMD Disease-Free Zone in Punjab Province, Pakistan
Source: Front Vet Sci. 2021 Aug 18;8:703473. doi: 10.3389/fvets.2021.703473 (PMC8416473; doi:10.3389/fvets.2021.703473)
Supplement: Supplementary Material A — Production system data. [file Data_Sheet_1.zip › Supplementary material E - Data request for beef feedlots - updated.pdf]

Supplementary material E – Data request for beef feedlots

Beef feedlot variable	Minimum	Most likely	Maximum
FMD outbreaks			
Morbidity rate (% of animals that get FMD during a typical outbreak)			
Case fatality rate (% of FMD cases that die)			
Extra days required for finishing in FMD cases (days)			
Treatment costs for case of FMD (PKR)			
Total time spent treating sick animals (minutes)			
Cost of additional vet services during outbreak (PKR)			
Feed costs			
Normal feed intakes per day (kg per animal)			
Feed intakes per day while animal has FMD (kg per animal)			
Cost per kg of feed			
Value of animals			
Value of finished animal (not affected by FMD) (PKR)			
Value of finished animal (if affected previously by FMD) (PKR)			
Other			
Labour costs (PKR per day)			
