# Supplementary material for: Economic Considerations for Advancement Through the Progressive Control Pathway: Cost–Benefit Analysis of an FMD Disease-Free Zone in Punjab Province, Pakistan
Source: Front Vet Sci. 2021 Aug 18;8:703473. doi: 10.3389/fvets.2021.703473 (PMC8416473; doi:10.3389/fvets.2021.703473)
Supplement: Supplementary Material A — Production system data. [file Data_Sheet_1.zip › Supplementary material F - Expert opinion exercise for desert livestock systems.pdf]

Supplementary Material F - Assumptions used in Cost-benefit analysis for FMD control in Pakistan

1. **Desert livestock systems**

In the February 2020 workshop, various parameters were estimated through expert to help estimate the production losses. During this time, no data were collected on the **desert livestock systems**. Their losses due to FMD need to be quantified so that all the benefits of control are fully incorporated. Some data were available in the literature but some assumptions were made and need expert validation.

For the parameters in the box below, estimated for cattle and buffalo separately, ***they were assumed to be the same as smallholder subsistence farms. If you disagree, please provide alternative estimates for desert livestock farms in Appendix A.***

- FMD incidence (% of animals with clinical FMD on an affected farm)
- Baseline mortality rate (% of animals dying each year on a normal farm)
- FMD case fatality rate (% of individual FMD cases that die)
- Offtake rates (% of animals normally sold each year from a farm)
- Culling rates (% of FMD cases that recover and are subsequently sold in the following year)
- Distress sales (% of FMD cases that are sold soon after becoming sick at a reduced value)
- Pregnancy rate (% of animals having a calf in a year)
- Heat detection rate (% of animals that are served to a heat)
- Voluntary waiting period (the number of days after calving that a cow is not served)
- Missed cycles in FMD cases (the number of oestrus cycles missed service due to FMD)
- Abortion rate in FMD cases
- Extended age of first calving in FMD cases (number of months)
- Value of animals (PKR per live animal by age-sex category)
- Proportion of cows lactating
- Cost of treatment (PKR per case of FMD)

The number of outbreaks in desert livestock farms needs to be estimated:
Key information to consider (provided by Dr Afzal)

- The estimated number of desert livestock farms in the zone is 19,000
- 1-2 outbreaks are reported each year but the true number of FMD outbreaks in desert livestock farms is unknown
- 2013 NSP seroprevalence study found all farms to have at least one positive animal;
- Overall individual animal seroprevalence was 55-62%
- Therefore NSP seroprevalence indicates massive under-reporting in this system

PLEASE COMPLETE THE FOLLOWING for number of outbreaks in desert livestock farms in the proposed zone (complete yellow boxes):

	Current estimate	Proposed new estimate
Minimum	10	
Most likely	100	
Maximum	1000	

The following sections refer to the costs required for entering PCP stage 3 and beyond in a single zone within Punjab province. The proposed zone is in Bahawalpur Division, an area which lends itself to enhanced FMD control my natural barriers to the east (Cholistan desert and border with India) and west (Sutlej river).<br><br>Numerous costs had to be assumed, which need to be checked by experts from Pakistan

2. Post-vaccination monitoring

Small-scale immunogenicity study

- Required to ensure vaccine quality for all vaccines used in the control zone and buffer area
- Performed according to FAO-OIE PVM guidelines
  - 12 seronegative animals are vaccinated using each vaccine used in the zone/buffer area
  - Samples are tested by VNT to each strain in the vaccine
- Need to estimate total cost to perform this test
  - All lab testing, field work costs to organise, keeping animals, and for each vaccine strain being used etc
- The total cost for this is estimated to be between 10,000 and 20,000USD (or 1.7-3.4 million PKR)

Small-scale immunogenicity studies	Current estimate (million PKR)	Proposed new estimate (million PKR)
Minimum	1.7	
Maximum	3.4	

3. **Surveillance activities**

The following parameters were assumed as part of estimating the costs of surveillance activities in the zone and buffer area. Please enter proposed new estimated in the yellow areas if you disagree with these estimates.

Surveillance activities	Category	Current estimate	Proposed new estimate
Cost of sending a single virus shipment to WRLFMD (PKR) including up to 50 samples	Minimum	300,000	
	Maximum	400,000	
Number of veterinary staff visiting each farm for taking samples when performing **outbreak investigation**	Fixed	2	
Number of veterinary staff visiting each farm for taking samples when performing **serosurveillance**	Fixed	2	
Number of farms visited per day during **serosurveillance**	Minimum	1	
	Most likely	3	
	Maximum	5	
Total number of full-time veterinary officers for market inspections, outbreak investigations, other surveillance activities	Fixed	15	

4. Sanitary measures

Parameter	Category	Current estimate	Proposed new estimate (***insert a range if you are uncertain***)
Number of quarantine stations required at border **between proposed zone and Sindh province**	Fixed	5	
Total cost of building a quarantine station*	Fixed	3.3 million PKR per station	
Annual maintenance cost for quarantine station**	Fixed	330,000 PKR per station per year	

*Cost of building a quarantine station assuming nothing exists currently and therefore should include ALL building costs and also connecting electricity and including computer facilities. If you are uncertain, you can include a range (minimum and maximum).
**Maintenance cost covers all bills and other miscellaneous expenses. If you are uncertain, you can include a range (minimum and maximum).

5. Programme management, stakeholder engagement and communications

Parameter	Category	Current estimate	Proposed new estimate
Number of senior managers*	Fixed	1	
Number of assistants **	Fixed	3	
Establishment of "Punjab Meat Board" (million PKR) ***	Minimum	0.85	
	Maximum	1.5	
Maintenance of "Punjab Meat Board" (million PKR per year) ****	Minimum	1.5	
	Maximum	2.8	
Stakeholder engagement and communications (initial costs in first two years of project) *****	Fixed	3.5	
Stakeholder engagement and communications (annual costs after first two years) *****	Fixed	2.0	

*Senior manager is in charge of running the zone and is employed full time for this purpose
** Assistants support the manager and manage implementation of the activities, and are also employed full time
*** These are initial costs to set up initial meetings and other activities related to the meat value chain, establishing the public-private partnership and exports
**** These are ongoing annual costs supporting all activities of the board
***** These are the costs required for all meetings with stakeholders including the production and dissemination of communication products (leaflets, television adverts etc). If you are uncertain, you can include a range (minimum and maximum).

6. Preparatory studies, training and capacity building

These are the costs of activities required for developing the control strategy and providing training as part of capacity building within Pakistan and the proposed zone.

Parameter	Category	Current estimate (million PKR)	Proposed new estimate (million PKR)
**Risk assessment and value chain analysis**<br>Initial studies are the cost of consultants to do the initial work and provide training to staff in Pakistan so that they can continue the yearly updates. Ongoing "refresher" training is provided every three years.			
- Initial studies	Minimum	8.0	
	Maximum	13.5	
- Yearly updates	Minimum	0.5	
	Maximum	0.85	
- Ongoing training	Minimum	0.85	
	Maximum	1.4	
**Surveillance and post-vaccination**<br>A single workshop for establish what is needed, with creating a plan for these activities			
- Workshops and plan development	Minimum	4.0	
	Maximum	7.0	
**Development of preparedness and contingency plans**<br>This is cost per workshop. Two workshops envisaged in total.			
- Workshops and plan development	Minimum	1.5	
	Maximum	3.0	
**Research project into role of small ruminants in FMD**			
- Grant or university (including all lab costs)	Minimum	5.0	
	Maximum	8.5	
**Outbreak investigation training**<br>Initial training from external consultants utilising a "train the trainer" approach so subsequent trainings (new staff and refresher trainings) are provided by staff in Pakistan			
- Initial field training course	Minimum	4.5	
	Maximum	5.5	
- Subsequent trainings (every two years)	Minimum	1.5	
	Maximum	3.0	
**Laboratory capacity development**<br>All training provided by external consultants			
- Training in VNT and vaccine matching	Minimum	1.5	
	Maximum	3.0	
- Establish QA system	Minimum	1.5	
	Maximum	3.0	
- Refresher training (every 3 years)	Minimum	0.85	
	Maximum	1.5	
- Participation in proficiency tests (yearly)	Minimum	0.85	
	Maximum	1.5	

Appendix A – Smallholder subsistence versus Desert Livestock farms

Note: you should only complete the tables for desert livestock systems if you disagree with the assumptions that the parameters is the same as smallholder subsistence farms.

Table 1a. Assumptions on desert livestock systems for estimating the costs of FMD. These are the parameters used for smallholder subsistence farmers and based on the study by Ferrari et al, 20130

Parameter	Age category	Smallholder subsistence	
		Buffalo	Cattle
FMD incidence (% of animals with clinical FMD on an affected farm)	<1 year	56%	70%
	Heifers and males >1 year old	50%	70%
	Cows	43%	60%

Table 1b. Alternative estimates for FMD incidence in **desert livestock systems** – ***only complete if you disagree with the assumption that it would be the same as smallholder subsistence farms. Note this is for unvaccinated farms.***

Parameter	Age category	Desert livestock farms					
		**Buffalo**			**Cattle**		
		Minimum	Most likely	Maximum	Minimum	Most likely	Maximum
FMD incidence (% of animals with clinical FMD on an affected farm)	<1 year						
	Heifers and males >1 year						
	Cows						

Table 2a. Estimates used for **smallholder subsistence farms** regarding baseline mortality and FMD case fatality rates

Parameter	Age-sex category	Smallholder subsistence					
		**Buffalo**			**Cattle**		
		Minimum	Most likely	Maximum	Minimum	Most likely	Maximum
Baseline mortality rate (% of animals dying each year on a normal farm)	Male calves <1 year	20%	28%	40%	10%	20%	30%
	Female calves <1 year	20%	28%	40%	10%	20%	30%
	Bulls > 1 year	5%	10%	15%	10%	10%	10%
	Heifers > 1 year	8%	13%	18%	10%	10%	10%
	Cows	2%	5%	9%	0%	10%	10%
FMD case fatality rate (% of individual FMD cases that die)	Male calves <1 year	18%	23%	40%	20%	20%	40%
	Female calves <1 year	18%	23%	40%	20%	30%	40%
	Bulls > 1 year	0%	2%	4%	0%	0%	0%
	Heifers > 1 year	0%	2%	4%	0%	0%	0%
	Cows	0%	0%	1%	0%	0%	0%

Table 2b. Estimates used for **desert livestock farms** regarding baseline mortality and FMD case fatality rates – ***<u>only complete if you disagree with the assumption that it would be the same as smallholder subsistence farms</u>*.**

Parameter	Age-sex category	Desert livestock farms					
		**Buffalo**			**Cattle**		
		Minimum	Most likely	Maximum	Minimum	Most likely	Maximum
Baseline mortality rate (% of animals dying each year on a normal farm)	Male calves <1 year						
	Female calves <1 year						
	Bulls > 1 year						
	Heifers > 1 year						
	Cows						
FMD case fatality rate (% of individual FMD cases that die)	Male calves <1 year						
	Female calves <1 year						
	Bulls > 1 year						
	Heifers > 1 year						
	Cows						

Table 3a. Estimates used for **smallholder subsistence farms** regarding baseline offtake rates, culling rates in FMD cases, and distress sales. These data are based on expert opinion collected during the February 2020 workshop in Islamabad.

Parameter	Age-sex category	Smallholder subsistence			
		**Buffalo**		**Cattle**	
		Minimum	Maximum	Minimum	Maximum
Offtake rates (% of animals normally sold each year from a farm)	Male calves <1 year	30%	60%	30%	60%
	Female calves <1 year	5%	9%	10%	10%
	Bulls > 1 year	65%	90%	70%	90%
	Heifers > 1 year	8%	18%	10%	20%
	Cows	3%	7%	0%	10%
Culling rates (% of FMD cases that recover and are subsequently sold in the following year)	Male calves <1 year	0%	10%	0%	10%
	Female calves <1 year	0%	10%	0%	10%
	Bulls > 1 year	0%	3%	0%	0%
	Heifers > 1 year	0%	3%	0%	0%
	Cows	4%	9%	0%	10%
Distress sales (% of FMD cases that are sold soon after becoming sick at a reduced value)	Male calves <1 year	0%	8%	0%	10%
	Female calves <1 year	0%	0%	0%	0%
	Bulls > 1 year	3%	29%	0%	3%
	Heifers > 1 year	0%	7%	0%	10%
	Cows	4%	9%	0%	10%

Table 3b. Estimates used for **desert livestock farms** regarding baseline offtake rates, culling rates in FMD cases and distress sales – ***only complete if you disagree with the assumption that it would be the same as smallholder subsistence farms.***

Parameter	Age-sex category	Desert livestock farms			
		**Buffalo**		**Cattle**	
		Minimum	Maximum	Minimum	Maximum
Offtake rates (% of animals normally sold each year from a farm)	Male calves <1 year				
	Female calves <1 year				
	Bulls > 1 year				
	Heifers > 1 year				
	Cows				
Culling rates (% of FMD cases that recover and are subsequently sold in the following year)	Male calves <1 year				
	Female calves <1 year				
	Bulls > 1 year				
	Heifers > 1 year				
	Cows				
Distress sales (% of FMD cases that are sold soon after becoming sick at a reduced value)	Male calves <1 year				
	Female calves <1 year				
	Bulls > 1 year				
	Heifers > 1 year				
	Cows				

Table 4a. Estimates used for **smallholder subsistence farms** regarding baseline fertility, abortion rates and the age of first calving. These data are based on expert opinion collected during the February 2020 workshop in Islamabad.

Parameter	Category	Smallholder subsistence			
		**Buffalo**		**Cattle**	
		Minimum	Maximum	Minimum	Maximum
Pregnancy rate (% of animals having a calf in a year)	Heifers	30%	60%	10%	70%
	Cows	35%	65%	40%	60%
Heat detection rate (% of animals that are served to a heat)	Heifers	46%	66%	40%	70%
	Cows	40%	58%	70%	60%
Voluntary waiting period (the number of days after calving that a cow is not served)	Cows only	90	147	60	110

Table 4b. Estimates used for **desert livestock farms** regarding baseline offtake rates, culling rates in FMD cases and distress sales – ***only complete if you disagree with the assumption that it would be the same as smallholder subsistence farms*.**

Parameter	Category	Desert livestock farms			
		**Buffalo**		**Cattle**	
		Minimum	Maximum	Minimum	Maximum
Pregnancy rate (% of animals having a calf in a year)	Heifers				
	Cows				
Heat detection rate (% of animals that are served to a heat)	Heifers				
	Cows				
Voluntary waiting period (the number of days after calving that a cow is not served)	Cows only				

<u>Table 5a.</u> Impact of FMD on fertility parameters used for **smallholder subsistence farms**. These data are based on expert opinion collected during the February 2020 workshop in Islamabad.

Parameter	Age-sex category	Smallholder subsistence					
		**Buffalo**			**Cattle**		
		Minimum	Most likely	Maximum	Minimum	Most likely	Maximum
Missed cycles in FMD cases (the number of oestrus cycles missed service due to FMD)	-	5	6.5	11	7.5	10.5	16
Abortion rate in FMD cases	Heifers	2	3%	8%	10%	10%	10%
	Cows	1%	3%	5%	10%	10%	10%
Extended age of first calving in FMD cases (number of months)	Heifers only	7	9	12	7.5	9	12.5

<u>Table 5b.</u> Estimates used for **desert livestock farms** regarding fertility parameters – ***<u>only complete if you disagree with the assumption that it would be the same as smallholder subsistence farms</u>.***

Parameter	Age-sex category	Desert livestock farms					
		**Buffalo**			**Cattle**		
		Minimum	Most likely	Maximum	Minimum	Most likely	Maximum
Missed cycles in FMD cases (the number of oestrus cycles missed service due to FMD)	-						
Abortion rate in FMD cases	Heifers						
	Cows						
Extended age of first calving in FMD cases (number of months)	Heifers only						

<u>Table 6a.</u> Value of cattle and buffalo by age and sex category for smallholder subsistence farms. These data are based on expert opinion collected during the February 2020 workshop in Islamabad. All values in PKR.

Age-sex category	Smallholder subsistence			
	**Buffalo**		**Cattle**	
	Minimum	Maximum	Minimum	Maximum
Female calves <1yo	10,500	17500	9,000	10,000
Male calves <1yo	12,500	22,500	8,000	9,000
Males >1yo	40,000	100,000	9,000	18,000
Heifer 1-2yo	65,000	85,000	95,000	160,000
Heifer 2-5 years old	85,000	170,000	70,000	90,000
Cows	110,000	225,000	130,000	230,000

<u>Table 6b.</u> Estimates used for **desert livestock farms** regarding values of cattle and buffalo by age and sex – ***<u>only complete if you disagree with the assumption that it would be the same as smallholder subsistence farms.</u>***

Age-sex category	Desert livestock farms			
	**Buffalo**		**Cattle**	
	Minimum	Maximum	Minimum	Maximum
Female calves <1yo				
Male calves <1yo				
Males >1yo				
Heifer 1-2yo				
Heifer 2-5 years old				
Cows				

Table 7a. Estimates used for estimating the proportion of cows that are lactating on a smallholder subsistence farm. These data are based on expert opinion collected during the February 2020 workshop in Islamabad. Note that other parameters for milk production are based on figures in the literature so expert opinion is not needed.

Parameter	Smallholder subsistence			
	**Buffalo**		**Cattle**	
	Minimum	Maximum	Minimum	Maximum
Proportion of cows lactating	40%	50%	40%	50%

Table 7b. Estimates used for **desert livestock farms** regarding the proportion of adult cows lacating – ***only complete if you disagree with the assumption that it would be the same as smallholder subsistence farms.***

Parameter	Desert livestock farms			
	**Buffalo**		**Cattle**	
	Minimum	Maximum	Minimum	Maximum
Proportion of cows lactating				

<u>Table 8a.</u> Treatment costs in buffalo and cattle assumed for smallholder subsistence farmers. These data are based on expert opinion collected during the February 2020 workshop in Islamabad. All prices in PKR.

Age-sex category	Smallholder subsistence					
	**Buffalo**			**Cattle**		
	Minimum	Most likely	Maximum	Minimum	Most likely	Maximum
Calves male and female < 1 year	2,500	3,500	4,500	2,500	3,500	4,500
Heifers and bulls < 1 year old	3,500	5,000	5,500	3,500	5,000	5,500

*Note for cows, the estimated cost was based on that reported by Shat et al, 2014: mean 1087, standard deviation 992.

<u>Table 8b.</u> Estimates used for **desert livestock farms** regarding treatment costs for FMD cases – ***<u>only complete if you disagree with the assumption that it would be the same as smallholder subsistence farms</u>.***

Age-sex category	Desert livestock farms					
	**Buffalo**			**Cattle**		
	Minimum	Most likely	Maximum	Minimum	Most likely	Maximum
Calves male and female < 1 year						
Heifers and bulls < 1 year old						
