# Supplementary material for: Economic Considerations for Advancement Through the Progressive Control Pathway: Cost–Benefit Analysis of an FMD Disease-Free Zone in Punjab Province, Pakistan
Source: Front Vet Sci. 2021 Aug 18;8:703473. doi: 10.3389/fvets.2021.703473 (PMC8416473; doi:10.3389/fvets.2021.703473)
Supplement: Supplementary Material A — Production system data. [file Data_Sheet_1.zip › Supplementary material H - Results - costs and benefits by year.pdf]

Supplementary material H

Costs and benefits of a proposed FMD disease free zone in Punjab province, Pakistan, over a 20-year period. Median, non-discounted values are presented. Benefits are based on averted production losses and treatment costs due to FMD alone. All values in million PKR.

Year of programme	Costs (billion PKR)	Benefits (billion PKR)
0	1.98	3.88
1	4.08	3.87
2	4.46	3.88
3	3.81	3.96
4	4.22	4.07
5	4.15	4.18
6	4.13	4.26
7	4.15	4.30
8	4.14	4.31
9	4.15	4.31
10	4.15	4.31
11	4.15	4.31
12	4.14	4.31
13	4.15	4.31
14	4.14	4.31
15	4.15	4.31
16	4.15	4.31
17	4.15	4.31
18	4.13	4.31
19	4.15	4.31
