# Supplementary figures and images for: Economic Considerations for Advancement Through the Progressive Control Pathway: Cost–Benefit Analysis of an FMD Disease-Free Zone in Punjab Province, Pakistan
Source: Front Vet Sci. 2021 Aug 18;8:703473. doi: 10.3389/fvets.2021.703473 (PMC8416473; doi:10.3389/fvets.2021.703473)

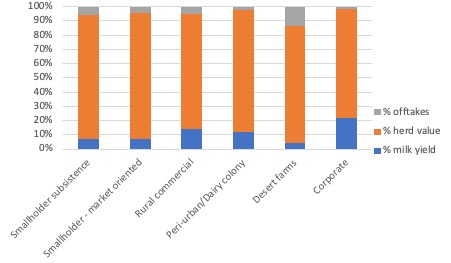

Supplement: Supplementary Material A — Production system data. [file Data_Sheet_1.zip › Supplementary material G - Breakdown of costs by production system.jpg]
